# Identification of dynamic undifferentiated cell states within the male germline

**DOI:** 10.1038/s41467-018-04827-z

**Published:** 2018-07-19

**Authors:** Hue M. La, Juho-Antti Mäkelä, Ai-Leen Chan, Fernando J. Rossello, Christian M. Nefzger, Julien M. D. Legrand, Mia De Seram, Jose M. Polo, Robin M. Hobbs

**Affiliations:** 1 Australian Regenerative Medicine Institute, Monash University, Melbourne, VIC 3800 Australia; 2 Development and Stem Cells Program, Monash Biomedicine Discovery Institute and Department of Anatomy and Developmental Biology, Monash University, Melbourne, VIC 3800 Australia; 3 Present Address: Institute of Biomedicine, Research Centre for Integrative Physiology and Pharmacology, University of Turku, Turku, Finland

## Abstract

The role of stem cells in tissue maintenance is appreciated and hierarchical models of stem cell self-renewal and differentiation often proposed. Stem cell activity in the male germline is restricted to undifferentiated A-type spermatogonia (A_undiff_); however, only a fraction of this population act as stem cells in undisturbed testis and A_undiff_ hierarchy remains contentious. Through newly developed compound reporter mice, here we define molecular signatures of self-renewing and differentiation-primed adult A_undiff_ fractions and dissect A_undiff_ heterogeneity by single-cell analysis. We uncover an unappreciated population within the self-renewing A_undiff_ fraction marked by expression of embryonic patterning genes and homeodomain transcription factor PDX1. Importantly, we find that PDX1 marks a population with potent stem cell capacity unique to mature, homeostatic testis and demonstrate dynamic interconversion between PDX1+ and PDX1− A_undiff_ states upon transplant and culture. We conclude that A_undiff_ exist in a series of dynamic cell states with distinct function and provide evidence that stability of such states is dictated by niche-derived cues.

## Abstract (web summary)

Sustained spermatogenesis depends on stem cell activity which is contained within a population of undifferentiated spermatogonia. Here, the authors identify a new population of undifferentiated spermatogonia in adult testis that expresses the transcription factor PDX1 and has stem cell capacity.

## Introduction

Limited lifespan of differentiated cells in many tissues necessitates replacement to ensure tissue maintenance. Within such high turnover tissues, resident stem cells generate fresh cohorts of differentiating cells. Stem cell populations can be heterogeneous and composed of multiple pools with distinct functional characteristics and involvement in tissue maintenance and repair^1^. Stem cell activity can also be context dependent^1^. The ability to define different stem cell subsets within a tissue is dependent on availability of molecular markers that delineate populations.

Sustained spermatogenesis and recovery of fertility following germ cell depletion are dependent on stem cells within the testis seminiferous epithelium^2^. In adult mice, germline stem cell activity is contained within a population of undifferentiated Type A spermatogonia (A_undiff_), which develop postnatally from foetal germ cells (gonocytes). Gonocytes migrate from the lumen of developing seminiferous tubules to the basement membrane and generate undifferentiated and differentiating spermatogonia. The adult A_undiff_ pool contains isolated single cells (A-single or A_s_) plus chains of cells remaining interconnected by cytoplasmic bridges after cell division; 2-cell chains are A-paired (A_pr_) while chains of 4 or more A-aligned (A_al_). A_undiff_ differentiation is marked by induction of c-KIT plus DNA methyltransferases 3A and 3B (DNMT3A/3B) and formation of A_1_ spermatogonia^3^. A_1_ cells undergo a series of mitotic divisions and via A_2_, A_3_, A_4_, Intermediate (In) and B spermatogonia generate meiotic spermatocytes. It is generally accepted that germline stem cell activity is contained within the A_undiff_ pool. However, A_undiff_ cells are heterogeneous and contrasting models of A_undiff_ hierarchy and stem cell identity are proposed^4,5^.

A founding model of spermatogonial hierarchy proposed that A_s_ act as stem cells while A_pr_ and A_al_, the bulk of A_undiff_, are differentiation-committed progenitors^2^. Upon division, A_s_ generate two A_s_ for self-renewal or A_pr_ that generate A_al_ and differentiate. This model has been challenged through transplantation and lineage tracing. Germline stem cells form long-lived spermatogenic colonies when injected into seminiferous tubules of germ cell-depleted recipients^6^. As each colony is normally derived from a single cell, stem cell numbers in the donor population can be estimated. While there are ~35,000 A_s_ in an adult testis, only ~3000 cells have transplant capability^7^, suggesting that not all A_s_ are stem cells. Recent studies concluded that transplant activity is almost exclusively contained within a fraction of A_s_ marked by *Id4* expression, supporting a revised A_s_ model in which stem cell activity is limited to a subset of A_s_ while remaining A_s_ plus A_pr_ and A_al_ are differentiation-committed or transiting into a committed state^5,8^. Given that dynamics of *Id4* expression are poorly understood and relationship between transplantation capacity and in situ stem cell activity is contentious^8,9^, it remains to be determined whether ID4+ A_s_ solely possess stem cell potential. Studies on ID4+ spermatogonia have often focused on neonates rather than adults^5,8^.

An alternative stem cell model is proposed based on in situ lineage tracing and live-cell imaging^4^. Namely, that stem cell potential is a dynamic property shared by most A_undiff_ irrespective of morphology but that gene expression dictates fate propensity. A_undiff_ expressing *Gfra1* (a co-receptor for glial cell line derived neurotrophic factor or GDNF) that are mostly A_s_, A_pr_ and some short A_al_, form an equipotent stem cell pool and transition between single cells and syncytial states^10^. A_undiff_ expressing Neurogenin3 (*Ngn3*), primarily A_al_ plus few A_s_ and A_pr_, are differentiation-destined due to expression of RARγ and sensitivity to the differentiation stimulus retinoic acid (RA)^4,11^. GFRα1− progenitors are also marked by transcription factor SOX3^12^. However, during testis regeneration or upon transplantation, NGN3+ cells contribute significantly to the stem cell pool^9^. NGN3+ A_al_ can fragment to shorter chains or A_s_ and revert to a GFRα1+ state, indicating that NGN3+ cells possess latent stem cell activity^4^. Further, subsets of NGN3+ cells marked by MIWI2 exhibit regenerative capacity but are dispensable for tissue homeostasis^13^. Whether stem cell potential is an inherent property of all A_undiff_ or restricted to a subset of cells remains to be confirmed. Regardless, stem cell activity in undisturbed tissue is primarily associated with the GFRα1+ pool while remaining A_undiff_ are differentiation-primed.

Niche-derived stimuli and intrinsically expressed factors regulate A_undiff_ self-renewal and differentiation. Supporting Sertoli cells produce GDNF, which is required for stem cell self-renewal^14^. Basic fibroblast growth factor (bFGF) is derived from a variety of cells and maintains A_undiff_ function in concert with GDNF^6^. Importantly, GDNF and bFGF support long-term culture of A_undiff_^6^. These factors promote A_undiff_ self-renewal via induction of cell cycle genes plus transcription factors BCL6B, ETV5 and LHX1^15–18^. Promyelocytic leukaemia zinc finger (PLZF or ZBTB16) promotes A_undiff_ self-renewal and is an A_undiff_ marker^19–21^. The transcription factor SALL4, a component of the pluripotency network of embryonic stem cells (ESCs) is expressed throughout the spermatogonial pool and essential for A_undiff_ differentiation and self-renewal^22,23^. Other pluripotency-associated factors such as *Oct4* (*Pou5f1*) are expressed in A_undiff_ and have functional roles^24^. While multiple genes are involved in A_undiff_ self-renewal and differentiation, our understanding of mechanisms underlying functional heterogeneity of A_undiff_ is limited.

Germline stem cell identity remains contentious largely due to our inadequate understanding of A_undiff_ heterogeneity and limited markers to discriminate functionally distinct A_undiff_ fractions. To address this heterogeneity at the molecular and functional level and shed light on spermatogonial hierarchy, we generated compound reporter mice based on *Plzf* and *Oct4* promoters that have distinct expression patterns within adult A_undiff_. These allowed us to isolate and define gene expression signatures of A_undiff_ fractions enriched in self-renewing and differentiation-primed cells. Based on this data and single cell analysis, we characterize A_undiff_ heterogeneity and identify a population within the GFRα1+ self-renewing pool marked by transcription factor PDX1 plus embryonic patterning genes. We find that PDX1 selectively marks a mature A_undiff_ population with potent transplantation capabilities and demonstrate an instructive role for niche factors in regulating interconversion between PDX1+ and PDX1− A_undiff_. Our studies indicate that A_undiff_ exist in a series of functionally distinct interconvertible states differentially stabilized through niche interactions.

## Results

### Generation of a Plzf reporter marking A_undiff_

**Fig. 1:**
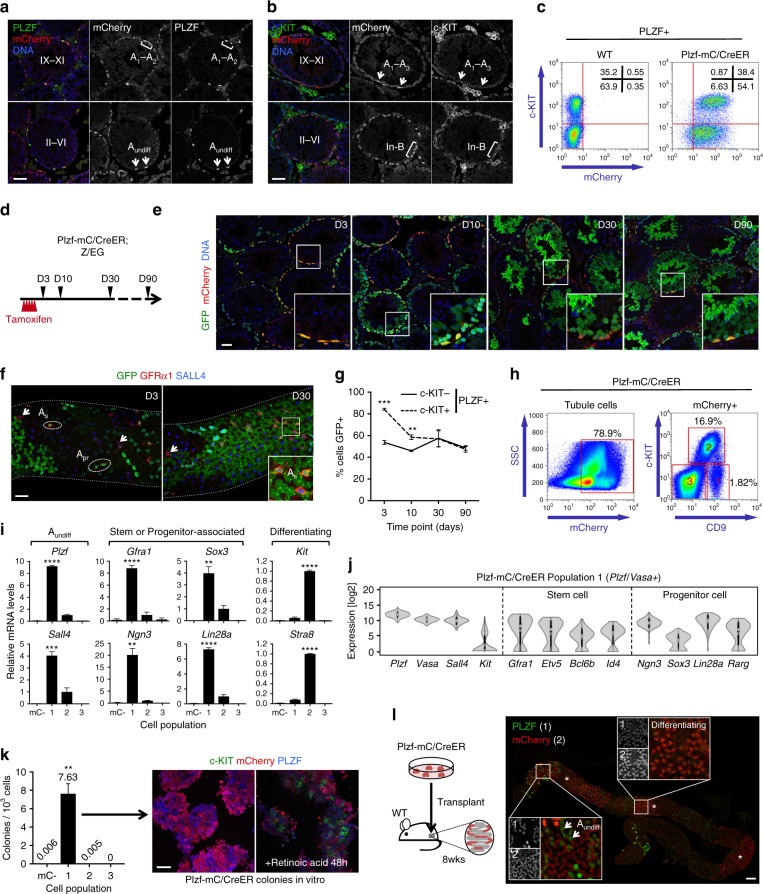
Characterization of Plzf-mC/CreER transgenic mice. **a**, **b** Representative IF of adult Plzf-mC/CreER testis sections (*n* = 3 mice). Tubule stages and populations are indicated. Scale bar, 50 μm. **c** Representative flow cytometry of fixed and permeabilized testis from Plzf-mC/CreER and wildtype (WT) adult testis (*n* = 3 mice per genotype). PLZF+ cells are shown. **d** Plzf-mC/CreER; Z/EG mice injected daily with TAM for 5 days were harvested at indicated days after treatment. **e** Representative IF of testis sections from **d** (*n* = 3 testes per time point). Insets show details of indicated areas. Scale bar, 50 μm. **f** Representative whole-mount IF from **d**. Inset shows detail of indicated area. Arrowheads: unlabelled GFRα1+ cells. Scale bar, 50 μm. **g** Flow cytometry of fixed and permeabilized testis cells from **d**. Graph indicates mean fraction of A_undiff_ (PLZF+ c-KIT−) and PLZF+ c-KIT+ early differentiating cells expressing GFP ± standard error of mean (s.e.m.) (*n* = 4 testes D3, D10 and D90, *n* = 6 testes D30). **h** Representative flow cytometry of live Plzf-mC/CreER testis cells. SSC is side scatter. mCherry+ gate was set according to WT. **i** Quantitative RT-PCR for spermatogonial markers from Plzf-mC/CreER cell fractions sorted as in **h**. mC− indicates mCherry−. Expression levels are corrected to β-actin and normalized so mean value of fraction #2 equals 1. Mean values ± s.e.m. are indicated (*n* = 3 sorts, 2 mice pooled per sort). Significance vs. mCherry− cells is shown. **j** Violin plots of gene expression in 150 single cells of fraction #1 cells from **h**. Cells were gated according to *Plzf* and *Vasa* expression. **k** Left: mean in vitro colony-forming activity of Plzf-mC/CreER fractions ± s.e.m. isolated as in **h** (*n* = 3 mice). mC− indicates mCherry−. Significance vs. mCherry− fraction is indicated. Right: representative IF of passaged cells from fraction #1 treated with vehicle or retinoic acid for 48 h (*n* = 3). Scale bar, 50 μm. **l** Left: transplantation of cultured cells established from Plzf-mC/CreER fraction #1. Right: representative whole-mount IF of tubules 8 weeks post transplant demonstrating formation of mCherry+ colonies (*n* = 5 recipients). Comparable spermatogenic capacity was observed upon transplantation of an independent line (3.90 colonies/10^5^ cells; *n* = 4 recipient testes). Scale bar, 100 μm. Significance was calculated by two-tailed Student’s *t*-test (**P < 0.01, ***P < 0.001, ****P < 0.0001)

Gene expression patterns characterizing A_undiff_ subsets remain poorly defined due to lack of markers allowing efficient A_undiff_ isolation. To develop a system for A_undiff_ purification, we generated transgenic mice expressing mCherry plus tamoxifen-regulated Cre from regulatory elements of *Plzf* (Plzf-mC/CreER) (Supplementary Fig. 1a)^19–21^. Immunofluorescence (IF) of testis sections demonstrated that PLZF+ spermatogonia were marked with mCherry in adults from two founders (Fig. 1a and Supplementary Fig. 1b). Similar to endogenous *Plzf*^20^, mCherry was prominent in A_undiff_ and differentiating type-A spermatogonia, identified according to expression of *Plzf* and *Kit* plus seminiferous epithelium cycle stage (Fig. 1a, b)^22^. mCherry was also detected in c-KIT+ spermatogonia at late differentiation stages (In/B) in which endogenous *Plzf* is downregulated (Fig. 1b). Increased stability of mCherry vs. PLZF or omission of regulatory elements in the transgene may account for this discrepancy. Analysis of fixed, permeabilized testis cells by flow cytometry demonstrated that ~90% of A_undiff_ (PLZF+ c-KIT−) and essentially all early differentiating cells (PLZF+ c-KIT+) expressed Plzf-mC/CreER (Fig. 1c and Supplementary Fig. 1c). Plzf-mC/CreER is an effective marker of undifferentiated and differentiating spermatogonia.

To confirm that Plzf-mC/CreER is expressed in stem cells, we crossed mice with a transgenic line expressing GFP upon Cre-mediated recombination (Z/EG)^25^. Tamoxifen (TAM) treatment of Plzf-mC/CreER; Z/EG adults induced GFP in mCherry+ spermatogonia within the epithelium basal layer by day 3 post-treatment (D3) (Fig. 1d, e). Some spermatocytes with low mCherry expression were also GFP+ . At later timepoints, GFP was expressed by mature spermatocytes then spermatids as progeny of transgene-expressing cells differentiated. The majority of tubule cross-sections contained GFP+ spermatogonia D90 after TAM (71.8% ± 1.06, *n* = 4 testes) (Fig. 1e). Given renewal of the seminiferous epithelium (one round of spermatogenesis takes ~35 days), retention of lineage-marked cells demonstrated that Plzf-mC/CreER was active in stem cells. Whole-mount IF demonstrated that GFP was induced and maintained in a substantial fraction of GFRα1+ A_s_ and A_pr_, consistent with stem cell expression (Fig. 1f)^10^. By flow cytometry ~50% of A_undiff_ (PLZF+ c-KIT−) were lineage-marked over the time course, indicating transgene activity in a stable self-renewing population (Fig. 1g and Supplementary Fig. 1d). Early differentiating cells (PLZF+ c-KIT+) labelled more efficiently than A_undiff_ at initial time points, reflecting prominent transgene expression in these cells (Fig. 1c), progression of marked cells to later differentiation stages and generation of new differentiating cohorts by labelled A_undiff_.

To isolate A_undiff_ from germ cells marked by Plzf-mC/CreER, we stained cells for CD9 and c-KIT, markers of stem and differentiating spermatogonia, respectively^3,26^. CD9 is also expressed by somatic cells but selection according to CD9 and Plzf-mC/CreER was predicted to allow A_undiff_ isolation while excluding somatic contaminants. mCherry+ cells could be divided into three fractions: CD9+ c-KIT− (#1), CD9+ c-KIT+ (#2) and CD9^low^ c-KIT− (#3) (Fig. 1h). Subset #1 expresses A_undiff_ markers (*Plzf*, *Sall4*) and markers of stem (*Gfra1*) and progenitor (*Ngn3*, *Sox3*, *Lin28a*) cells and corresponds to the A_undiff_ population (Fig. 1i). Subset #2 expresses *Kit* and *Stra8*, suggesting it contains differentiating spermatogonia and early spermatocytes^3,27^. Cells in fraction #3 were presumably mature spermatocytes that contained low mCherry levels (Fig. 1a, b). Selection of cells with high mCherry excluded population #3 while #1 and #2 contained cells with a range of mCherry expression (Supplementary Fig. 1e). A_undiff_ could not be isolated using CD9 and c-KIT alone due to contamination with Plzf-mC/CreER− somatic cells (Supplementary Fig. 1f).

To confirm identity and purity of this putative A_undiff_ fraction, we analysed gene expression of population #1 at a single cell level by qRT-PCR (Fig. 1j). 150 out of 152 cells were positive for expression of *Plzf* and germ cell marker *Vasa*, indicating somatic cell exclusion and A_undiff_ enrichment. These cells homogenously expressed *Sall4* while *Kit* expression was limited to few cells. Stem cell-associated genes *Gfra1*, *Etv5*, *Bcl6b* and *Id4* exhibited bimodal expression, consistent with heterogeneous expression in A_undiff_. Progenitor-associated genes *Sox3* and *Rarg* also displayed bimodal expression patterns. The ability to generate stable clusters of cells in vitro was essentially limited to fraction #1 and cultured cells expressed PLZF, GFRα1 and mCherry, resembling A_undiff_ (Fig. 1k and Supplementary Fig. 1g)^20^. Cultured cells upregulated c-KIT plus STRA8 and downregulated A_undiff_ markers in response to RA, confirming differentiation competence (Fig. 1k and Supplementary Fig. 1g)^24^. Cultures generated colonies upon transplantation, confirming stem cell activity (Fig. 1l). Through development of a *Plzf* reporter, we therefore established a system for A_undiff_ isolation.

### Pluripotency reporters exhibit distinct expression in adult A_undiff_

**Fig. 2:**
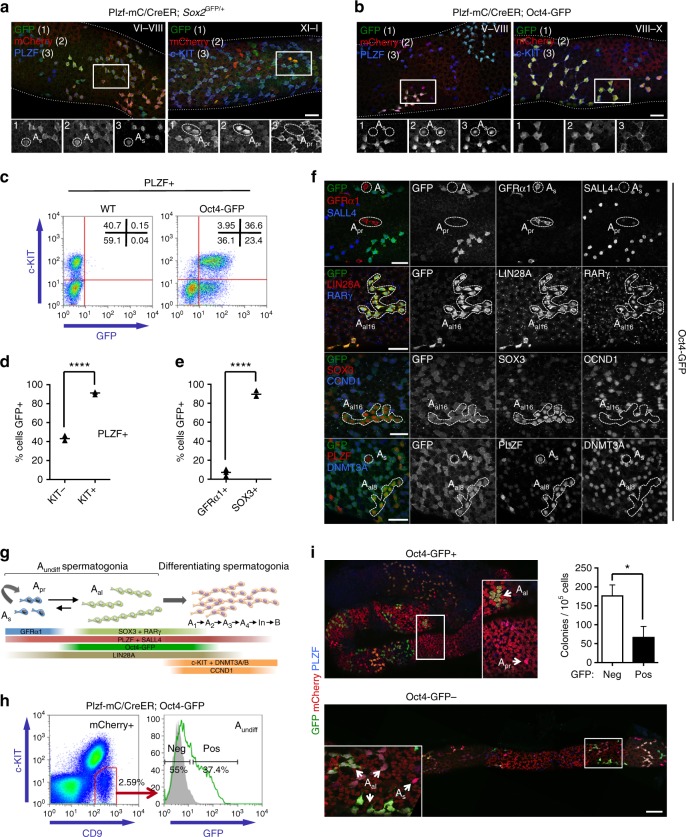
Comparative analysis of reporter gene expression in spermatogonia. **a**, **b** Representative whole-mount IF of adult (8–10 weeks post natal) Plzf-mC/CreER; *Sox2*^GFP^ (**a**) and Plzf-mC/CreER; Oct4-GFP (**b**) seminiferous tubules. Inset panels show individual immunostaining within indicated area at higher magnification. Tubule staging and select A_s_ and A_pr_ are indicated. Scale bars, 50 μm. **c** Representative flow cytometry analysis of fixed and permeabilized testis cells from 1 of 3 Oct4-GFP and wild-type (WT) control adults. PLZF+ cell population is shown. Percentages of cells contained within gates are indicated. **d** Quantification of flow cytometry results from **c**. Graph indicates percentage of A_undiff_ (PLZF+ c-KIT−) and cells initiating differentiation (PLZF+ c-KIT+) expressing GFP in Oct4-GFP adults. Horizontal bars indicate mean values (*n* = 3 mice). **e** Graph shows percentage of GFRα1+ and SOX3+ spermatogonia positive for GFP in whole-mount seminiferous tubules of Oct4-GFP adults. Spermatogonial identity was confirmed by SALL4 counterstain. Horizontal bars indicate mean values (*n* = 3 mice, >200 cells scored per data point). **f** Representative whole-mount IF of adult Oct4-GFP seminiferous tubules for indicated markers (*n* = 3 mice). Select A_undiff_ cells are indicated. Scale bar, 50 μm. **g** Scheme summarizing expression patterns of indicated genes and transgenic reporters plus changes in cell morphology during spermatogonial differentiation. Markers used to isolate different spermatogonial populations are indicated. **h** Isolation of Oct4-GFP− and Oct4-GFP+ A_undiff_ from Plzf-mC/CreER; Oct4-GFP adults by flow cytometry. Percentage of cells in each gate from a representative sample is indicated (*n* = 6 mice). **i** Oct4-GFP− and GFP+ adult A_undiff_ fractions were transplanted into recipients and analysed 8 weeks later by whole-mount IF. Images show GFP and mCherry expression in representative colonies. PLZF counterstain confirms A_undiff_ and spermatogonial identity. Panels show higher magnification details of indicated areas. Scale bar, 100 μm. Graph shows colony-forming efficiency of Oct4-GFP+ and GFP− A_undiff_ fractions. Data is presented as mean number of colonies per 10^5^ donor cells ± s.e.m. (*n* = 7 recipient testes for Oct4-GFP− cells and *n* = 6 for Oct4-GFP+ cells). Donor cells were pooled from 2 Plzf-mC/CreER; Oct4-GFP adults. Significance was calculated by two-tailed Student’s *t*-test (*P < 0.05, ****P < 0.0001)

To isolate functionally distinct A_undiff_ and characterize heterogeneity, we developed compound reporter mice with Plzf-mC/CreER, which is expressed throughout the A_undiff_ pool, and GFP-based reporters marking discrete subsets. Pluripotency gene reporters are associated with stem cell activity and potentially exhibit heterogeneous expression within A_undiff_^28,29^. *Sox2*^GFP^ marks transplantable testis cells and lineage-marked cells of *Sox2*^CreER^ adults generate long-lived clones, demonstrating stem cell expression^28^. Restriction of *Sox2*^GFP^ expression to isolated spermatogonia in sections suggested that *Sox2*^GFP^ marks A_s_ and short A_undiff_^28^. However, whole-mount IF demonstrated that Plzf-mC/CreER and *Sox2*^GFP^ were co-expressed throughout the A_undiff_ pool and in c-KIT+ differentiating spermatogonia (Fig. 2a). *Oct4* reporters mark transplantable cells in neonatal testis, suggesting expression in stem cells^30,31^. The transgene here is based on a 18 kb *Oct4* gene fragment lacking proximal enhancer sequences and displays heterogeneous expression in cultured A_undiff_^29,32^. Whole-mount IF of adult Plzf-mC/CreER; Oct4-GFP tubules indicated that Oct4-GFP expression was limited to a subset of Plzf-mC/CreER+ spermatogonia (Fig. 2b). Surprisingly, while Oct4-GFP was expressed in A-type differentiating spermatogonia and A_al_, it was generally absent from Plzf-mC/CreER+ A_s_ and A_pr_ (Fig. 2b). Analysis of A_undiff_ by intracellular staining and flow cytometry confirmed heterogeneous Oct4-GFP expression (Fig. 2c). ~40% of PLZF+ c-KIT− A_undiff_ and ~90% of PLZF+ c-KIT+ were Oct4-GFP+ (Fig. 2d), revealing a correlation between Oct4-GFP and differentiation.

In agreement with these results, GFRα1+ A_s_ and A_pr_ (steady-state stem cells^10^) were generally Oct4-GFP− (Fig. 2e, f). In contrast, most SOX3+ progenitors (including A_al_ and A_1_/A_2_) were Oct4-GFP+ (Fig. 2e, f). We confirmed that Oct4-GFP was preferentially expressed in SOX3+ progenitors vs. GFRα1+ A_undiff_ at all stages of the epithelium cycle (Supplementary Fig. 2a,b). Oct4-GFP expression positively correlated with spermatogonial chain length in both populations (Supplementary Fig. 2c). By comparison to markers of progenitors (RARγ, LIN28A) and differentiating cells (DNMT3A, CCND1)^3,20^, we confirmed that Oct4-GFP was expressed in A_al_ and differentiating A-type cells and downregulated at later differentiation stages (Fig. 2f, g).

Gonocytes of postnatal day 2 (PND2) testes were Oct4-GFP+ as anticipated, confirming transgene integrity (Supplementary Fig. 2d)^32^. By PND5 Oct4-GFP was heterogeneously expressed in PLZF+ spermatogonia and GFRα1+ cells had lower Oct4-GFP expression than GFRα1− cells suggesting that Oct4-GFP expression is suppressed as gonocytes migrate into the niche. OCT4 protein was restricted to Oct4-GFP+ cells in the neonate (Supplementary Fig. 2d), indicating that Oct4-GFP recapitulates aspects of endogenous *Oct4* expression.

To test function of Oct4-GFP+ and Oct4-GFP− A_undiff_, cells from Oct4-GFP; Plzf-mC/CreER adults were transplanted and mCherry+ colonies scored after 8 weeks (Fig. 2h). While stem cell activity was significantly enriched within the Oct4-GFP− A_undiff_ fraction as expected, the Oct4-GFP+ population also contained transplantation activity (Fig. 2i). Oct4-GFP therefore delineates A_undiff_ populations with distinct characteristics and stem cell activity but transplantation capability is not restricted to the Oct4-GFP− population^9,13^.

### Gene expression signatures of stem and progenitor A_undiff_

**Fig. 3:**
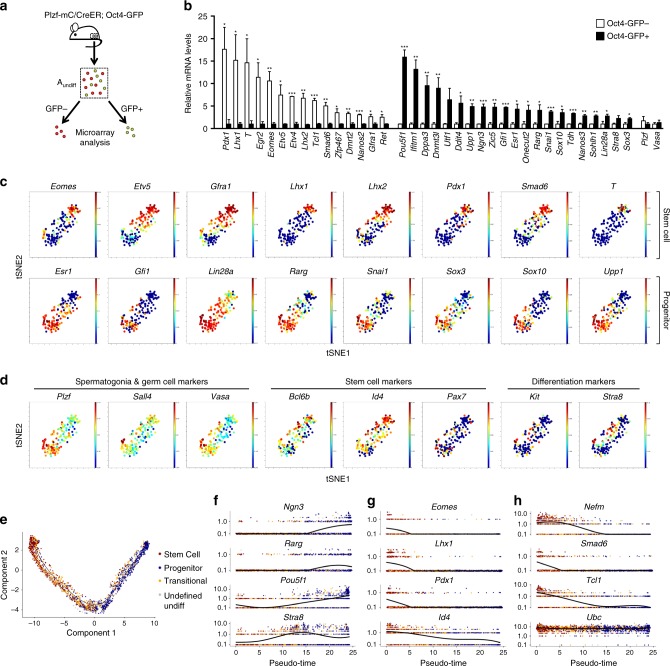
Identification and characterization of distinct A_undiff_ populations. **a** Oct4-GFP− and Oct4-GFP+ A_undiff_ fractions were isolated from Plzf-mC/CreER; Oct4-GFP adults for gene expression profiling by microarray. A_undiff_ fraction is mCherry+ CD9+ c-KIT−. **b** Confirmation of gene expression signatures of Oct4-GFP− and Oct4-GFP+ A_undiff_ by quantitative RT-PCR. Candidate genes were selected from microarray analysis of **a**. Expression levels are corrected to those of β-actin and normalized so mean value of GFP− or GFP+ fractions equals 1. Mean values from 3-5 mice ± s.e.m. are indicated. Genes enriched in Oct4-GFP− and Oct4-GFP+ populations are shown in separate groups. Control genes *Plzf* and *Vasa* are shown. Significance was calculated by two-tailed Student’s *t*-test (*P < 0.05, **P < 0.01, ***P < 0.001). **c** viSNE maps derived from single cell analysis of 150 single A_undiff_ cells isolated from pooled adult Plzf-mC/CreER testis. Based on expression of a total of 71 genes identified from analysis of A_undiff_ fractions in **b** plus genes previously linked with A_undiff_ function. Relative expression of selected genes associated with stem and progenitor fractions by each plotted cell is indicated (red = high, blue = low). **d** viSNE maps from single cell analysis of **c**. Spermatogonial and germ cell markers are in left panels. Middle panels show stem cell markers not identified as differentially expressed in Oct4-GFP− and GFP+ A_undiff_ fractions by microarray. Differentiation markers are in right panels. **e** A_undiff_ isolated from 2 Plzf-mC/CreER; Oct4-GFP adults were analysed by single cell RNA-Seq and developmental trajectory calculated using Monocle. Cells were classified as follows: Stem cells (*Gfra1* or *Etv5* positive), progenitors (*Sox3* or *Upp1* positive), transitional cells (positive for combinations of stem and progenitor genes) and undefined undiff (negative for these stem and progenitor genes). **f**−**h** Expression of indicated genes across cell trajectory (pseudotime) from single cell analysis of A_undiff_ in **e**. Expression of housekeeping gene *Ubc* is shown

To gain insight into A_undiff_ heterogeneity we performed gene expression profiling of Oct4-GFP− and GFP+ A_undiff_ (Fig. 3a and Supplementary Data 1). From a panel of differentially expressed genes selected according to potential relevance for stem cell function, we confirmed gene expression signatures of Oct4-GFP− and GFP+ A_undiff_ by qRT-PCR (Fig. 3b). Oct4-GFP+ A_undiff_ expressed progenitor markers (*Ngn3*, *Rarg*, *Nanos3*, *Sohlh1*, *Lin28a*, *Stra8*, *Sox3*) at a higher level than Oct4-GFP− A_undiff_. Genes downstream GDNF and/or linked with stem cell function (*Lhx1*, *T*, *Egr2*, *Etv5*, *Nanos2*, *Gfra1*, *Ret*) were enriched in Oct4-GFP− cells^16,33^. Expression of stem cell marker *Id4* was modestly although not significantly enriched in GFP− A_undiff_ (not shown). Control genes *Plzf* and *Vasa* were comparably expressed (Fig. 3b). These data support the observed expression pattern of Oct4-GFP in A_undiff_ and reveal gene signatures associated with stem and differentiation-primed (progenitor) fractions. Genes associated with pluripotency and embryonic germ cells were co-enriched with *Oct4* in the GFP+ progenitor fraction (*Dppa2*, *Dppa3*, *Gdf3*, *Lin28a*, *Prdm14*, *Tdh*, *Utf1*) (Fig. 3b and Supplementary Data 1). Expression of multiple developmental and cell regulators (*Eomes*, *Lhx1*, *Pdx1*, *Smad6*, *T*, *Tcl1*) were enriched within the GFP− fraction.

To confirm heterogeneous expression of genes from our screen, we analysed candidates plus known A_undiff_ and germ cell markers by single cell qRT-PCR of A_undiff_ from Plzf-mC/CreER adults. Data were processed with viSNE, which plots cells in a two-dimensional map where proximity of cell points indicates gene expression similarity^34^. Projection of expression of a gene of interest in colour allows visualization of cell subsets marked by that gene (Fig. 3c and Supplementary Fig. 2e). Spermatogonia with highest expression of stem cell-associated genes including *Gfra1* grouped top right of the viSNE map while cells with highest expression of progenitor-associated genes including *Rarg* and *Sox3* clustered at the opposing end. Cells in between these two groups co-expressed select stem and progenitor genes suggesting they represented transitional states. Expression of genes broadly active in A_undiff_ (*Plzf*, *Sall4*, *Vasa*) was found throughout the plot (Fig. 3d). While expression of stem cell genes *Bcl6b* and *Id4* was most evident within the stem cell fraction, they were detected in progenitors (Fig. 3d). *Pax7* was expressed sporadically (Fig. 3d)^35^. Expression of differentiation markers *Kit* and *Stra8* was detected, particularly in transitional cells (Fig. 3d).

Heterogeneity was apparent within the *Gfra1*+ stem cell population; expression of *Etv5* and *Lhx2* overlapped extensively with *Gfra1*, while some appeared restricted to a subset. Developmental regulators *Eomes*, *Pdx1*, *T* and *Lhx1* marked a subset of *Gfra1*-expressing cells at one extremity of the viSNE map furthest from progenitors, indicating that gene expression was distinct (Fig. 3c).

Unexpectedly, *Oct4* expression was present throughout the viSNE plot, contrasting with restricted expression of Oct4-GFP in progenitors (Supplementary Fig. 2e). The Oct4-GFP reporter marks *Oct4* expression in the embryonic germline but lacks specific enhancer elements and is randomly integrated^32^. Effects of integration site on transgene activity or regulatory roles of omitted enhancers might underlie differences in Oct4-GFP and *Oct4* expression. However, expression of *Oct4* and other pluripotency genes were similarly enriched in Oct4-GFP+ A_undiff_, supporting reporter validity (Fig. 3b). Differences in analysis methods, reporter mouse backgrounds or expression of *Oct4* elements retained in the Oct4-GFP reporter may underlie these discrepancies^32^.

To confirm expression of genes of interest including *Oct4* at a single cell level, we isolated A_undiff_ from Plzf-mC/CreER; Oct4-GFP mice for single-cell RNA-Seq. Data were processed using Monocle to organize cells in a predicted developmental trajectory (pseudotime) according to transcriptional similarities^36^. After quality controls we identified ~3500 germ cells. Trajectory calculations were performed in semi-supervised mode using expression of genes associated with stem (*Gfra1*, *Etv5*) and progenitor (*Sox3*, *Upp1*) populations (Fig. 3c) to identify the developmental pathway. A trajectory was obtained from cells only expressing one or both stem markers to those only expressing one or both progenitor markers (Fig. 3e). Transitional cells were defined as expressing combinations of stem and progenitor markers and were distributed along the trajectory with tendency to concentrate mid-trajectory (Fig. 3e). A minor population not identified as stem or progenitor cells according to these markers were “undefined undifferentiated”. Expression of progenitor genes *Ngn3* and *Rarg* increased as cells progressed along the trajectory, indicating that it recapitulated stem to progenitor transition (Fig. 3f). *Oct4* expression increased at late pseudotime in agreement with Oct4-GFP expression in progenitors (Fig. 3f). *Stra8* expression increased mid-pseudotime, confirming transient induction upon stem cell commitment (Fig. 3d, f). Expression of developmental regulators *Eomes*, *Lhx1* and *Pdx1* was limited to initial trajectory stages, consistent with stem cell expression (Fig. 3c, g). *T* expression was below the set detection level. *Id4* was expressed throughout the trajectory but with increased levels at early points indicating preferential but not exclusive stem cell expression (Fig. 3d, g). Other stem cell-associated genes expressed in early trajectory were confirmed, e.g., *Nefm*, *Smad6* and *Tcl1* (Fig. 3h). Examination of differentially expressed genes along the trajectory revealed three clusters corresponding to genes downregulated (stem cell-associated), upregulated (progenitor-related) or transiently upregulated across pseudotime (Supplementary Fig. 3). This latter cluster included cell cycle-related genes potentially involved in stem-progenitor transition. We therefore define gene expression signatures of distinct A_undiff_ populations and dynamics of gene expression during stem-to-progenitor conversion.

### Identification of a unique A_undiff_ population

**Fig. 4:**
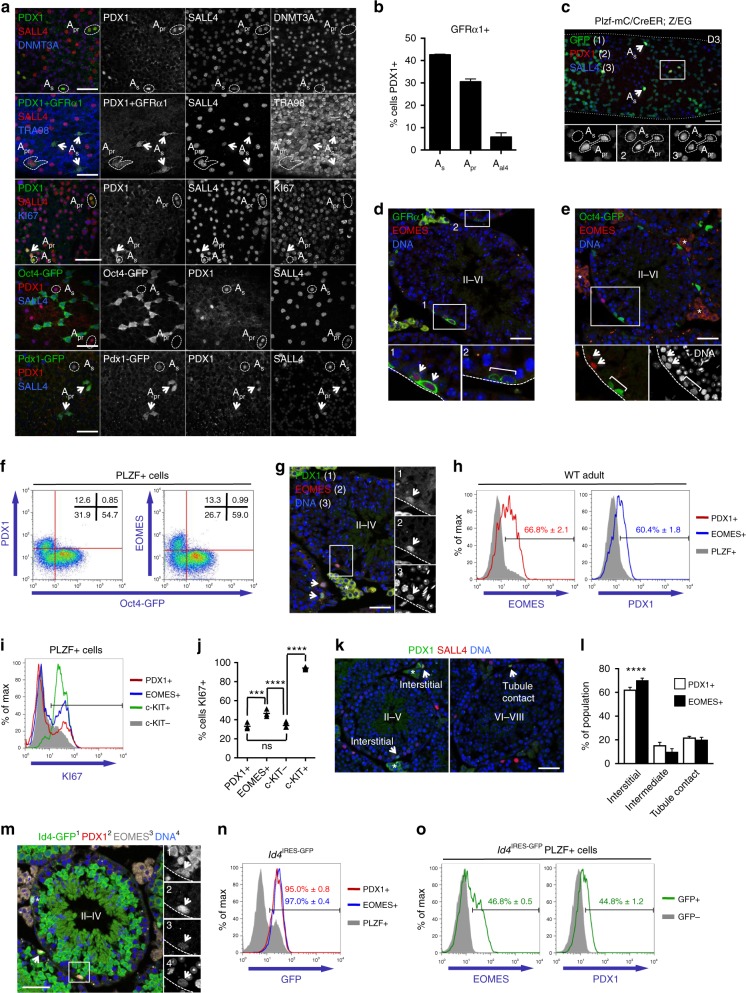
Characterization of PDX1+ spermatogonia. **a** Representative whole-mount IF of WT (top 3 rows, *n* = 3 mice), Oct4-GFP and *Pdx1*^GFP^ adult tubules (*n* = 2 mice per genotype). Antibodies to PDX1 and GFRα1 are goat polyclonals and distinguished by nuclear *vs*. cell surface staining respectively. Scale bars, 50 μm. **b** Mean percentage of GFRα1+ cells/chains PDX1+ ± s.e.m. from WT analysis in **a** (*n* = 4 mice, >250 cells/chains per sample). **c** Representative whole-mount IF of adult Plzf-mC/CreER; Z/EG tubules D3 post-TAM (*n* = 2 mice). Arrowheads: PDX1+ A_s_. Inset shows detail of indicated area. Scale bar, 50 μm. **d** Representative IF of adult WT testis sections (*n* = 4 mice). Insets: details of selected areas. EOMES+ GFRα1+ (arrowheads) and EOMES− GFRα1+ cells (bracket) are shown. Tubule stage is indicated. Scale bar, 50 μm. **e** Representative IF of adult Oct4-GFP testis section (*n* = 2 mice). Insets: immunostaining within indicated area. EOMES+ GFP− (arrowheads) and EOMES− GFP+ (bracket) spermatogonia are indicated. Asterisks: autofluorescent interstitium. Scale bar, 50 μm. **f** Representative flow cytometry of fixed and permeabilized testis cells from Oct4-GFP adults (*n* = 2). PLZF+ cells shown. **g** Representative IF of adult WT testis sections (*n* = 4 mice). Insets: immunostaining within indicated area. PDX1+ EOMES+ spermatogonia are indicated (arrowheads). Tubule stage is shown. Asterisk: autofluorescent interstitium. Scale bar, 50 μm. **h** Representative flow cytometry of fixed and permeabilized WT adult testis cells (*n* = 4). Mean numbers of EOMES+ and PDX1+ cells in PDX1+ and EOMES+ gates respectively are shown ± s.e.m. **i** Representative flow cytometry for KI67 in indicated PLZF+ populations of fixed and permeabilized adult WT testis cells (*n* = 4 mice). **j** Quantification of flow cytometry from **i**. Percentage of indicated PLZF+ fractions KI67+ . Horizontal bars: mean values (*n* = 4 mice). **k** Representative IF of adult WT testis sections demonstrating PDX1+ A_undiff_ localisation (arrowheads) within tubules (*n* = 4 mice). Asterisks: autofluorescent interstitium. Tubule stages are shown. Scale bar, 50 μm. **l** Quantification of spermatogonial localisation from **k**. Mean values ± s.e.m. are shown (*n* = 4 mice, 56–95 tubule sections per mouse). Significance vs. tubule-tubule localisation is indicated. **m** Representative IF of *Id4*^IRES-GFP^ adult testis sections (*n* = 4 mice). Insets show immunostaining within indicated area. Arrowheads: PDX1+ EOMES+ GFP+ spermatogonia. Asterisk: PDX1^low^ EOMES+ GFP+ cell. Tubule stage is shown. Scale bar, 50 μm. **n** Representative flow cytometry of indicated PLZF+ populations of fixed and permeabilized adult *Id4*^IRES-GFP^ testis cells (*n* = 4 mice). Mean numbers of PDX1+ and EOMES+ A_undiff_ expressing GFP ± s.e.m. **o** Flow cytometry of *Id4*^IRES-GFP^ testis cells as in **n**. PLZF+ fractions are shown. Mean numbers of PLZF+ GFP+ cells positive for EOMES and PDX1 are indicated ± s.e.m. (*n* = 4 mice). Significance was calculated by two-tailed Student’s *t*-test (***P < 0.001, ****P < 0.0001)

Single cell analysis revealed a subset of A_undiff_ within the GFRα1+ self-renewing pool that co-expressed *Pdx1*, *Eomes*, *T* and *Lhx1*. To confirm existence of this population, we assessed *Pdx1* expression in adults by whole-mount IF (Fig. 4a). *Pdx1* encodes a homeodomain transcription factor with roles in pancreas development^37^. Nuclear PDX1 was observed in SALL4+ DNMT3A− isolated spermatogonia and two cell-chains, indicating expression in A_s_ and A_pr_. Flow cytometry of fixed and permeabilized testis cells demonstrated that PDX1+ cells represent a minor fraction (~15%) of PLZF+ spermatogonia and are c-KIT−, confirming A_undiff_ identity (Supplementary Fig. 4a, b). PDX1+ cells were present at all stages of the seminiferous epithelium and a proportion was KI67+ suggesting that PDX1 does not mark A_undiff_ with unique mitotic status (Fig. 4a and Supplementary Fig. 4c)^2^. Consistent with single cell data, PDX1 was restricted to a subset of GFRα1+ A_undiff_ (Fig. 4a). ~30-40% of GFRα1+ A_s_ and A_pr_ were PDX1+ while GFRα1+ A_al_ were typically PDX1− (Fig. 4b). PDX1+ cells were Oct4-GFP− (Fig. 4a). In testis from *Pdx1*^GFP^ adults^38^, GFP was detected in PDX1+ A_s_ and A_pr_, demonstrating antibody specificity (Fig. 4a and Supplementary Fig. 4d). Importantly, the Plzf-mC/CreER reporter was active in PDX1+ cells in a lineage-tracing setting (Fig. 4c).

We next sought to confirm additional markers of PDX1+ A_undiff_. While LHX1 and T antibodies performed inadequately, we detected the T-box transcription factor EOMES in ~50% of GFRα1+ spermatogonia in adult testis sections (Fig. 4d and Supplementary Fig. 4e). By flow cytometry, EOMES+ cells were within the PLZF+ c-KIT− A_undiff_ fraction and comprised ~15% of PLZF+ spermatogonia (Supplementary Fig. 4f, g). EOMES+ cells were Oct4-GFP− and lineage-marked in Plzf-mC/CreER; Z/EG mice upon TAM (Fig. 4e and Supplementary Fig. 4h). Flow cytometry confirmed that PDX1+ and EOMES+ cells were only present within the Oct4-GFP− fraction of the PLZF+ population (Fig. 4f). While PDX1+ spermatogonia were frequently EOMES+, *Pdx1* and *Eomes* expression did not overlap completely; ~60-65% of PDX1+ cells were EOMES+ and vice versa (Fig. 4g, h). That PDX1 and EOMES delineated overlapping but distinct populations was evident from mitotic status. While percentage of PDX1+ cells positive for proliferation marker KI67 was similar to the bulk A_undiff_ pool, a higher proportion of EOMES+ cells were KI67+ (Fig. 4i, j). Both cell types were present throughout the seminiferous epithelium cycle but found more frequently at early stages (Supplementary Fig. 4i). Moreover, both populations localised preferentially to tubule regions adjacent to the interstitium (Fig. 4k, l)^39^.

To confirm the immature nature of PDX1+ and EOMES+ A_undiff_ we assessed expression of stem cell marker *Id4* in this population using *Id4*^IRES-GFP^ mice^5,40^. *Id4* reporter expression in adults was prominent in spermatocytes plus spermatids and marked occasional spermatogonia (Fig. 4m and Supplementary Fig. 4j). While most GFP+ cells were PLZF− c-KIT− by flow cytometry (spermatocytes and spermatids) a subset were PLZF+ c-KIT− (A_undiff_) (Supplementary Fig. 4k). Approximately 30% of PLZF+ spermatogonia expressed *Id4*^IRES-GFP^ (Supplementary Fig. 4l, m). Spermatogonia co-expressing *Pdx1*, *Eomes* and *Id4* were observed in sections but not all ID4+ spermatogonia were PDX1+ or EOMES+ (Fig. 4m and Supplementary Fig. 4j). By flow cytometry, essentially all PDX1+ and EOMES+ cells were ID4+ but only ~45% of ID4+ spermatogonia were PDX1+ or EOMES+ (Fig. 4n, o), confirming more widespread expression of *Id4* than reported^5^. Combined, we identify *Id4*-expressing A_undiff_ marked by PDX1 and EOMES within the GFRα1+ stem cell pool.

### Functional and molecular features of PDX1+ spermatogonia

**Fig. 5:**
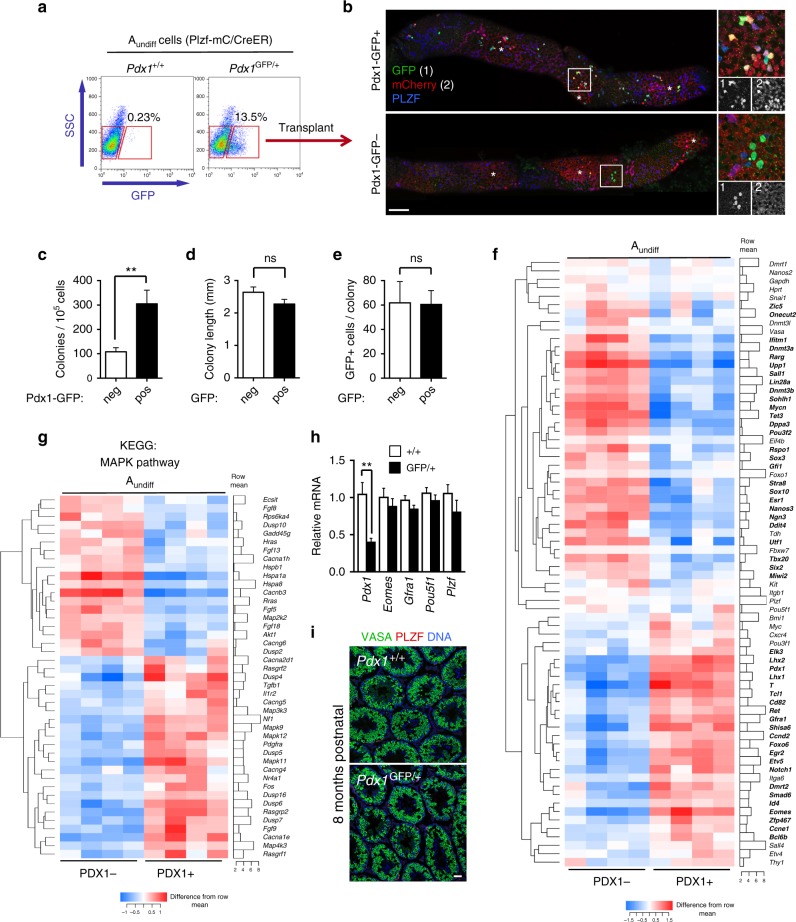
Stem cell potential and molecular characteristics of PDX1+ A_undiff_. **a** Isolation of PDX1+ and PDX1− A_undiff_ from Plzf-mC/CreER; *Pdx1*^GFP/+^ adults by flow cytometry. A_undiff_ are mCherry+ CD9+ c-KIT−. Gates for GFP+ and GFP− cells were set according to Plzf-mC/CreER control (left profile). Percentage of cells in GFP+ gate from representative sample is shown (*n* = 7). **b** Pdx1-GFP+ and GFP− adult A_undiff_ fractions were transplanted into recipient testis and analysed 8 weeks later by whole-mount IF. Images show GFP and mCherry expression in representative donor colonies. Panels on right show higher magnification details of indicated areas and grayscale panels show individual immunostaining. Scale bar, 100 μm. **c** Colony-forming efficiency of Pdx1-GFP+ and GFP− A_undiff_ fractions in transplantation assays from **b**. Data is presented as mean number of colonies per 10^5^ donor cells ± s.e.m. (*n* = 15 recipient testes for Pdx1-GFP− cells and *n* = 13 for Pdx1-GFP+ cells). Donor cells were pooled from a total of 7 Plzf-mC/CreER; *Pdx1*^GFP/+^ adults. **d** Mean length ± s.e.m. of donor colonies was measured from tiled microscope images from experiment of **c**. **e** Mean number of GFP+ cells/donor colony ± s.e.m. were calculated for a set of whole-mount samples from transplant assay of **c** (*n* = 58 colonies from Pdx1-GFP− cells and *n* = 63 from Pdx1-GFP+). **f** Heatmap illustrates expression of indicated genes from RNA-Seq analysis of Pdx1-GFP+ and GFP− A_undiff_ fractions isolated as in **a** (*n* = 4 mice). Differentially expressed genes (DEG) are in bold. Cut-off for DEG is false discovery rate (FDR) < 0.05 and absolute fold change ≥1.5. **g** Heatmap showing differentially expressed MAPK pathway genes from KEGG analysis of RNA-Seq data from **f**. **h** Quantitative RT-PCR analysis of indicated genes in mCherry+ CD9+ c-KIT− A_undiff_ isolated from Plzf-mC/CreER; *Pdx1*^GFP/+^ and Plzf-mC/CreER; *Pdx1*^+/+^ control adults. Mean values ± s.e.m. are shown (*n* = 6 mice per genotype). **i** Representative IF of testis sections from aged (8 months) mice of the indicated genotypes (*n* = 3 mice). VASA and PLZF staining identifies germ cell and spermatogonial populations respectively. Scale bar, 50 μm. Significance was calculated by two-tailed Student’s *t*-test (**P < 0.01, not significant (ns) *P* > 0.05)

*Pdx1* expression is detected in GFRα1+ A_s_ and A_pr_, suggesting it marks stem cells. To test function of PDX1+ cells, GFP+ and GFP− A_undiff_ from *Pdx1*^GFP^; Plzf-mC/CreER mice were transplanted and mCherry+ colonies scored 8 weeks later. A small subset of mCherry+ CD9+ c-KIT− A_undiff_ was GFP+ (Fig. 5a). While both GFP+ and GFP− fractions generated colonies, PDX1+ cells exhibited significantly higher colony-forming activity than PDX1− A_undiff_ (Fig. 5b, c). Stem cell potential is therefore substantially enriched in but not exclusive to the PDX1+ population. Colony size from PDX1+ and PDX1− fractions was comparable (Fig. 5d), indicating that PDX1+ and PDX1− stem cells are equipotent. Equivalent GFP+ populations were found in colonies from both fractions, demonstrating that PDX1− A_undiff_ generate PDX1+ cells upon transplantation and vice versa (Fig. 5b, e).

To define molecular features of PDX1+ cells, we isolated GFP+ and GFP− A_undiff_ from *Pdx1*^GFP^; Plzf-mC/CreER adults for RNA-Seq. Expression of stem cell-associated genes identified from previous analysis (Fig. 3b), including *Egr2*, *Eomes*, *Etv5*, *Gfra1*, *Lhx1*, *Lhx2*, *Pdx1*, *Smad6*, *T* and *Tcl1* were enriched in the PDX1+ population (Fig. 5f). Genes associated with progenitors including *Dppa3*, *Esr1*, *Lin28a*, *Ngn3*, *Rarg*, *Sohlh1*, *Sox3*, *Sox10* and *Upp1* were expressed at higher levels in PDX1− cells (Fig. 5f). Control genes *Plzf*, *Sall4*, and *Vasa* were comparably expressed while *Id4* was expressed at a higher level in PDX1+ cells. Wnt pathway inhibitor *Shisa6* was enriched in the PDX1+ fraction^41^. *Oct4* was not differentially expressed between PDX1+ and PDX1− fractions, consistent with discrepancies between endogenous *Oct4* and Oct4-GFP expression.

KEGG pathway analysis was performed on genes differentially expressed between PDX1+ and PDX1− A_undiff_ (Supplementary Data 2). Significant pathways included protein synthesis (ribosome, ribosome biogenesis), metabolism (OXPHOS) and cellular signalling (MAPK and NOTCH pathways) (Supplementary Fig. 5a). PDX1+ cells expressed lower levels of ribosome subunits than PDX1− A_undiff_ (Supplementary Fig. 5b), consistent with a need to limit protein synthesis rates^42^. MAPK pathway components (*Map2k2*/*Mek2*, *Map3k3*/*Mekk3*, *Mapk9*/*Jnk2* and *Mapk11*/*p38b*) plus MAPK regulators (*Hras*, *Nf1*, *Dusp2*, *Dusp4*, *Dusp5*, *Dusp6* and *Dusp7*) were differentially expressed in PDX1+ and PDX1− A_undiff_ (Fig. 5g), suggesting a central role for this pathway in A_undiff_ regulation^15,43^.

The *Pdx1*^GFP^ reporter disrupts *Pdx1* expression due to GFP insertion^38^. *Pdx1* loss is fatal due to defective pancreas development, so the reporter was maintained in heterozygosity^44^. Given reported haploinsufficiency of *Pdx1*^45^ and reduced *Pdx1* expression in *Pdx1*^GFP/+^ A_undiff_ (Fig. 5h and Supplementary Fig. 5c, d), we assessed whether germline maintenance was disrupted in *Pdx1*^GFP/+^ adults. No disruptions to PLZF+ and differentiating c-KIT+ populations were evident in aged *Pdx1*^GFP/+^ mice (Fig. 5i and Supplementary Fig. 5e). Expression of stem cell-associated genes *Eomes* and *Gfra1* in A_undiff_ from *Pdx1*^GFP/+^ adults was not perturbed (Fig. 5h). PDX1 therefore marks A_undiff_ with unique molecular features and potent stem cell capabilities but reduction in *Pdx1* expression does not compromise stem cell function.

### Niche factors control transitions between A_undiff_ states

**Fig. 6:**
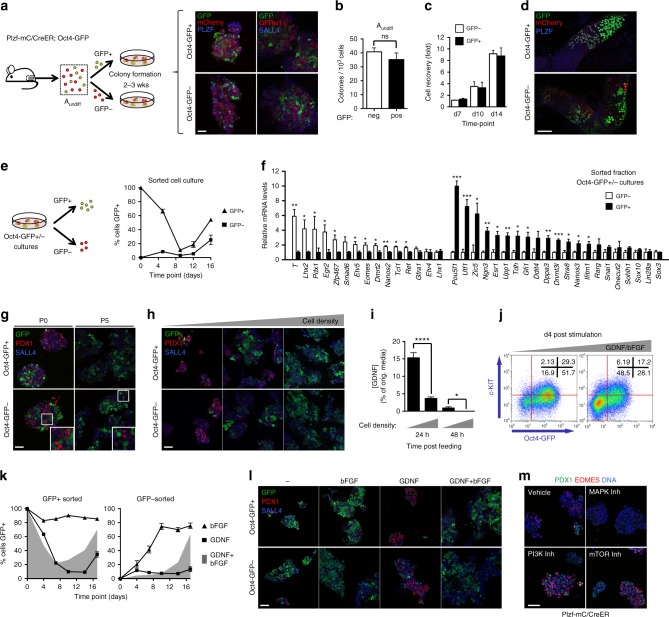
A_undiff_ heterogeneity during culture. **a** Oct4-GFP− and GFP+ A_undiff_ from Plzf-mC/CreER; Oct4-GFP adults placed in culture and analysed 2–3 weeks later. Right: IF of colonies (*n* = 2 per fraction). Scale bar, 50 μm. **b** Mean colony-forming efficiency of Oct4-GFP− and GFP+ A_undiff_ ± s.e.m. from **a** (*n* = 6 mice). **c** Cultures from Oct4-GFP− and GFP+ A_undiff_ plated at 25 × 10^3^ per well and counted at indicated timepoints. Mean recovery ± s.e.m. shown. **d** Cultures from Oct4-GFP− and GFP+ A_undiff_ transplanted and analysed 8 weeks later by IF. Representative colonies shown (2 sets of lines) (*n* = 11 testes Oct4-GFP− and *n* = 9 testes Oct4-GFP+). Scale bar, 100 μm. **e** Cultures from Oct4-GFP− and GFP+ A_undiff_ sorted by GFP and plated at 25 × 10^3^ per well. GFP+ cells determined by flow cytometry. Mean ± s.e.m. shown (*n* = 6 cultures). **f** qRT-PCR of GFP+ and GFP− cells from Oct4-GFP− and GFP+ A_undiff_ cultures. Expression corrected to β-actin and normalized so mean of GFP− or GFP+ fractions equals 1. Mean ± s.e.m. shown (*n* = 4 cultures). **g** Representative IF of primary colonies (P0) and passage 5 (P5) cultures from Oct4-GFP− and GFP+ A_undiff_ (*n* = 4 lines). Scale bar, 50 μm. **h** Cultures from Oct4-GFP− and GFP+ A_undiff_ plated at increasing densities (10 × 10^3^, 100 × 10^3^ and 200 × 10^3^ cells/well) and analysed 7–10 days later. Representative IF shown (*n* = 4 lines). Scale bar, 50 μm. **i** Cultures from Oct4-GFP− and GFP+ A_undiff_ plated at low and high densities (20 × 10^3^, 200 × 10^3^ cells/well) and cultured for 2 weeks. Conditioned media was collected at indicated times (hours) after media replenishment for ELISA. Mean GDNF levels are shown as percentage of starting levels ± s.e.m. (*n* = 4 cultures). **j** Cultures from Oct4-GFP− A_undiff_ (20 × 10^3^ cells/well) switched to media containing reduced GDNF and bFGF (1 ng/ml, left) or maintained with regular media (right) for 4 days. Representative flow cytometry shown. **k** Cultures from Oct4-GFP− and GFP+ A_undiff_ sorted according to GFP and plated (10 × 10^3^ cells/well) in media with GDNF but no bFGF (GDNF) or bFGF without GDNF (bFGF). Cells analysed at indicated timepoints by flow cytometry. Mean percentage of cells GFP+ ± standard deviation (s.d.) (*n* = 3 replicates) from representative experiment shown. Grey plots: mean values in regular media (GDNF+ bFGF). **l** Cultures from Oct4-GFP− and GFP+ A_undiff_ (20 × 10^3^ cells/well) grown 3 days in regular media switched to media without GDNF or bFGF (−), bFGF without GDNF (bFGF), GDNF without bFGF (GDNF) or regular medium (GDNF+ bFGF) then analysed by IF after 2 weeks. Representative images shown (*n* = 2 lines). Scale bar, 50 μm. **m** Plzf-mC/CreER cultures incubated in media containing indicated inhibitors (Inh) for 4 days prior to IF. Representative images shown (*n* = 2 lines). Scale bar, 50 μm. Two-tailed Student’s *t*-test used (*P < 0.05, **P < 0.01, ***P < 0.001, ****P < 0.0001)

To examine hierarchy of A_undiff_ fractions and mechanisms controlling fate, we used our compound reporter models and culture system. When stem and progenitor-enriched A_undiff_ fractions were isolated from Plzf-mC/CreER; Oct4-GFP adults according to GFP and placed in culture, comparable numbers of cell clusters were formed (Fig. 6a, b). Cultures derived from Oct4-GFP− and GFP+ A_undiff_ had similar growth rates (Fig. 6c). Established lines were positive for A_undiff_ markers (PLZF, SALL4, GFRα1) plus mCherry and expressed Oct4-GFP heterogeneously (Fig. 6a). Our data suggested that cultures can be initiated from self-renewing and differentiation-primed A_undiff_ and cells readily interconvert between stem and progenitor states in vitro (Fig. 2i). Cultures from both sources generated colonies upon transplantation, confirming stem cell capacity (Fig. 6d and Supplementary Fig. 6a).

To confirm dynamics of A_undiff_ interconversion, sorted Oct4-GFP− and GFP+ cells from established lines were plated at defined density and GFP expression monitored (Fig. 6e). The majority of Oct4-GFP+ cells transitioned into a GFP− state within 1 week but GFP+ cells accumulated at later points. Oct4-GFP− cells generated GFP+ populations over a 2-week period. Adoption of a GFP+ state correlated with increasing culture density (Supplementary Fig. 6b).

Gene expression signatures of freshly isolated Oct4-GFP− and GFP+ A_undiff_ were mostly retained in fractions from cultures, indicating that Oct4-GFP marks similar populations in vitro and in vivo (Fig. 6f). Importantly, expression of stem cell-associated markers *Egr2*, *Eomes*, *Etv5*, *Lhx2*, *Pdx1* and *T* were enriched in Oct4-GFP− cells in vitro. However, *Lhx1* became evenly expressed between Oct4-GFP− and GFP+ fractions in culture (Fig. 6f). To confirm abundance of A_undiff_ fractions during culture, we compared *Pdx1* and Oct4-GFP expression upon passaging (Fig. 6g). Cell clusters formed from both stem and progenitor-enriched A_undiff_ fractions contained PDX1+ and Oct4-GFP+ cells. *Pdx1* and Oct4-GFP expression were mutually exclusive and *Eomes* expression mirrored that of *Pdx1* (Supplementary Fig. 6c). Oct4-GFP+ cells dominated upon passaging while PDX1+ and EOMES+ cells became relatively rare (Fig. 6g and Supplementary Fig. 6c).

As cultures are founded at clonal density then passaged at higher densities, we hypothesized that declining abundance of PDX1+ and EOMES+ cells upon passaging was due to high-density culture. When plated at low density, established cultures contained prominent PDX1+ and EOMES+ populations and reduced numbers of Oct4-GFP+ cells while increasing density caused accumulation of Oct4-GFP+ cells and reduced PDX1+ and EOMES+ populations (Fig. 6h and Supplementary Fig. 6d). Importantly, GDNF was more rapidly exhausted from media of cells cultured at high vs. low density and increasing GDNF and bFGF levels reduced abundance of Oct4-GFP+ cells and c-KIT+ differentiating populations (Fig. 6i, j and Supplementary Fig. 6e).

These data suggested an instructive role for niche factors in promoting stem vs. progenitor cell states. As GDNF and bFGF regulate self-renewal via distinct pathways^46^, we assessed importance of each factor in maintenance of the stem cell state. From sorted Oct4-GFP+ cells of established cultures, GDNF promoted adoption of an Oct4-GFP− state while bFGF did not (Fig. 6k). Conversely, the Oct4-GFP− state of sorted cells was lost when bFGF alone was present but maintained with GDNF (Fig. 6k). Cell growth under single factor conditions was more limited than with both factors (Supplementary Fig. 6f). When maintained with bFGF alone, cultures contained more c-KIT+ cells, consistent with switch to a differentiation-primed state (Supplementary Fig. 6g). GDNF thus plays a dominant role in supporting the stem cell fraction. Cell clusters formed with GDNF alone contained more PDX1+ cells than those in media containing bFGF alone (Fig. 6l). When in an appropriate environment, A_undiff_ therefore reversibly transition between progenitor and stem cell states. Interconversion between discrete states is regulated by niche factors.

Consistent with the role of ERK MAPK downstream GDNF in vivo^43^, treatment with a MAPK pathway inhibitor depleted PDX1+ and EOMES+ populations in low-density cultures and increased Oct4-GFP expression (Fig. 6m and Supplementary Fig. 6h, i). Inhibitors to PI3Kinase and mTOR, both linked to A_undiff_ differentiation^43,47,48^, promoted generation of EOMES+ cells and suppressed proliferation as indicated by KI67 (Fig. 6m and Supplementary Fig. 6i). Effects of PI3Kinase and mTOR inhibitors on PDX1+ populations were less pronounced (Fig. 6m and Supplementary Fig. 6i), suggesting that regulatory inputs to these genes are distinct.

### PDX1 and EOMES define distinct stem cell states

**Fig. 7:**
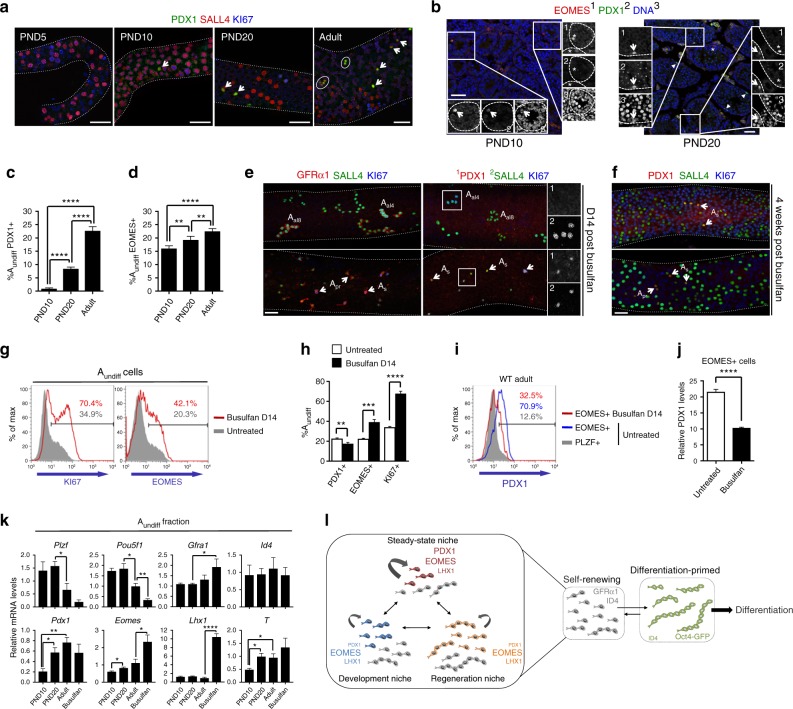
PDX1+ and EOMES+ spermatogonia during development and regeneration. **a** Representative whole-mount IF of tubules from WT mice of indicated ages (PND; postnatal day) (*n* = 2 mice per age). Scale bars, 50 μm. **b** Representative IF of testis sections from WT mice of indicated ages (*n* = 3 mice per timepoint). Insets show details of indicated areas. Arrowheads: EOMES+ PDX1+ cells. Asterisks: EOMES+ PDX1− cells. Scale bars, 50 μm. **c**, **d** Flow cytometry of fixed and permeabilized testis cells from WT mice of indicated ages. Mean percentages of PLZF+ c-KIT− A_undiff_ expressing PDX1 and EOMES are shown ± s.e.m. (*n* = 3–5 mice/time point). **e** WT adults were treated with busulfan (10 mg/kg) and tubules analysed by whole-mount IF 14 days later (*n* = 2 mice). Top panels: regenerative areas with GFRα1+ A_al_. Bottom: non-regenerative areas lacking GFRα1+ A_al_. Insets show details of indicated regions. Scale bar, 50 μm. **f** Regeneration assay of **e** 4 weeks post busulfan (2 areas shown). Arrowheads: PDX1+ A_undiff_. Scale bar, 50 μm. **g** Representative flow cytometry of fixed and permeabilized testis cells from WT adults untreated or busulfan treated as in **e** (*n* = 4 busulfan-treated mice and *n* = 5 untreated). PLZF+ c-KIT− A_undiff_ are shown. Percentage of A_undiff_ KI67+ and EOMES+ are indicated. **h** Mean percentages of PLZF+ c-KIT− A_undiff_ expressing PDX1, EOMES and KI67 from **g** are shown ± s.e.m. **i** Representative flow cytometry of PDX1 in indicated populations from **g** (*n* = 4 mice per condition). Percentages of cells PDX1+ are indicated. **j** PDX1 levels (median fluorescent intensity) in EOMES+ PLZF+ cells from **i**. Mean values ± s.e.m. shown. **k** Quantitative RT-PCR of A_undiff_ from Plzf-mC/CreER mice of indicated ages or 10 days post busulfan as in **e**. Expression levels are corrected to β-actin and normalized to an adult sample. Mean values ± s.e.m. are indicated (*n* = 4 PND10 and busulfan-treated, *n* = 5 PND20, *n* = 6 adults). Selected significance values are shown. **l** Model of A_undiff_ functional states. Self-renewing (curved arrows) GFRα1+ A_undiff_ adopt different states identified by variable expression of *Pdx1*, *Eomes* and *Lhx1*. A fraction of GFRα1+ A_undiff_ lacks PDX1, EOMES and LHX1 (grey in panel) and may represent a transitional state destined to become differentiation-primed. Niche signals differentially support self-renewing states. Given dynamic niche properties, different states predominate in development and homeostatic plus regenerative testis. The state marked by PDX1, EOMES and LHX1 is specific to homeostatic testis and potentially optimized for life-long germline maintenance. *Pdx1* is downregulated and *Eomes* plus *Lhx1* upregulated in states suited for short-term expansion during development and regeneration. Oct4-GFP+ differentiation-primed A_undiff_ convert back to a self-renewing state under optimised culture conditions or by transplantation into recipient testis with vacant niches. Significance was calculated by two-tailed Student’s *t*-test (*P < 0.05, **P < 0.01, ***P < 0.001, ****P < 0.0001)

The nascent stem cell population is formed during the first postnatal week of testis development and expands dramatically until steady-state spermatogenesis is established^49,50^. PDX1+ cells were not found in neonatal testis (PND5) and were most evident in adults (Fig. 7a). Occasional PDX1+ cells were observed at PND10 but significant populations not present until PND20. In contrast, EOMES was detectable in subsets of GFRα1+ spermatogonia at PND10 and PND20 (Fig. 7b and Supplementary Fig. 7a). At PND10, EOMES+ cells were generally PDX1− although some were PDX1^low^. EOMES+ PDX1+ cells became evident by PND20 (Fig. 7b). Distinct developmental timelines for PDX1+ and EOMES+ A_undiff_ were confirmed by flow cytometry (Fig. 7c, d and Supplementary Fig. 7b, c).

*Pdx1* induction coincides with transition from growing to steady-state tissue, indicating that PDX1 marks mature stem cells. Consistent with distinct regulatory inputs, EOMES marks stem cells of developing and mature testis although the proportion of EOMES+ A_undiff_ increased modestly during development (Fig. 7d). The proportion of A_undiff_ and EOMES+ cells that were KI67+ declined from PND10 to PND20 and adults consistent with declining mitotic status of stem cells (Supplementary Fig. 7d-f)^51^. *Pdx1* expression did not correlate with A_undiff_ quiescence (Fig. 4i, j), suggesting that PDX1 is not responsible for lower proliferative rates of mature stem cells.

Male germline cells are depleted by the alkylating agent busulfan. Regeneration of the seminiferous epithelium is reliant on “repopulating stem cells” resistant to genotoxic stress but whether these cells represent a specific A_undiff_ subset remains unclear^52^. To test whether PDX1 marks repopulating stem cells, WT mice were treated with a low busulfan dose that depletes differentiating spermatogonia and a substantial A_undiff_ fraction (Supplementary Fig. 7g-i)^4,22^. Few spermatogonia one-week after treatment expressed *Pdx1*, suggesting that PDX1 does not mark busulfan-resistant A_undiff_ (Supplementary Fig. 7j). By 2 weeks, remaining A_undiff_ have initiated a regenerative response characterized by long GFRα1+ A_al_ rarely observed in undisturbed testis (Fig. 7e)^4^. These A_al_ were mitotically active as indicated by KI67 but were PDX1−. PDX1+ cells were evident in areas containing GFRα1+ A_s_ and A_pr_ but few A_al_, which did not appear actively regenerating (Fig. 7e). At 4 weeks, tubule regions contained dense populations of A_undiff_ and differentiating spermatogonia, indicating successful regeneration (Fig. 7f). PDX1+ populations were found within these areas, consistent with PDX1 marking steady-state stem cells.

Analysis of A_undiff_ 2 weeks post busulfan by flow cytometry revealed that coincident with increased mitotic activity indicated by KI67, a greater proportion were EOMES+ (Fig. 7g, h). *Eomes* expression was still restricted to GFRα1+ cells during regeneration (Supplementary Fig. 7k). While most EOMES+ A_undiff_ express *Pdx1* in steady-state testis (Fig. 4h), PDX1 levels in EOMES+ cells under regenerative conditions were reduced (Fig. 7i, j). Accordingly, the proportion of A_undiff_ expressing *Pdx1* was lower than in controls (Fig. 7e, h). Our data suggest that stem cells upregulate *Eomes* and downregulate *Pdx1* under regenerative conditions. Alternatively, EOMES+ A_undiff_ might be busulfan-resistant and downregulate *Pdx1* during regeneration.

To confirm changes in gene expression during development and regeneration, we isolated A_undiff_ from Plzf-mC/CreER mice at different postnatal ages and 10 days after busulfan for qRT-PCR (Fig. 7k and Supplementary Fig. 7l, m). Across postnatal ages (PND10, 20, adult), expression of stem markers *Gfra1* and *Id4* were essentially constant while *Gfra1* modestly increased after busulfan (Fig. 7k). *Pdx1* expression was increased in A_undiff_ from PND20 and adults compared to PND10 while expression was modestly although not significantly downregulated after busulfan, reflecting persistence of PDX1+ cells in some tubules (Fig. 7e). As anticipated, *Eomes* expression increased marginally during development but was strongly upregulated after busulfan (Fig. 7k). Other genes marking PDX1/EOMES+ adult A_undiff_ exhibited distinct dynamics. *Lhx1* expression was constant during development but strikingly (~10 fold) increased following busulfan. *T* expression increased during development but was less responsive to busulfan. Control genes *Plzf* and *Oct4*/*Pou5f1* were downregulated in adult *vs*. PND20 A_undiff_ and reduced following busulfan, suggesting prominent roles in developing rather than steady state or regenerative A_undiff_. Changes in *Plzf* expression were reflected in altered activity of the Plzf-mC/CreER reporter (Supplementary Fig. 7n). Distinct combinations of *Eomes*, *Lhx1*, *Pdx1* and *T* expression therefore mark stem cells of developing, homeostatic and regenerating testis.

## Discussion

A_undiff_ heterogeneity is poorly characterized and cellular hierarchy debated. Taking advantage of compound reporter mice and single cell analysis, we defined gene expression signatures associated with distinct A_undiff_ populations. We confirmed that A_undiff_ are broadly divided into GFRα1+ (stem) and RARγ+ (differentiation-primed) fractions^10,11^. However, gene expression was not homogenous within these populations and we identify a subset of GFRα1+ cells marked by co-expression of *Pdx1*, *Eomes*, *Lhx1* and *T*. Heterogeneous *T* expression within GFRα1+ spermatogonia is reported but functional relevance incompletely understood^41^. We demonstrated by transplantation that PDX1+ cells have significantly enhanced stem cell activity compared to remaining A_undiff_ but that stem cells are not exclusive to this fraction. Moreover, in vivo and in vitro evidence indicates that A_undiff_ reversibly transition between these discrete populations and implicate niche factors in regulation of such transitions. *Pdx1* induction within A_undiff_ coincided with testis maturity while other genes marking adult PDX1+ cells, e.g., *Eomes*, were expressed during testis development. Further, *Pdx1*, *Lhx1* and *Eomes* displayed distinct expression dynamics during regeneration. We therefore propose a revised model of A_undiff_ hierarchy, whereby adult A_undiff_ exist in a series of dynamic interconvertible states that variably express *Pdx1*, *Eomes*, *Lhx1*, *Gfra1* and Oct4-GFP and have distinct functional characteristics (Fig. 7l). A_undiff_ states are stabilized through interactions with a cognate niche, the property of which varies during development, regeneration and upon tissue homeostasis. Niche status dictates whether specific states, e.g., PDX1+, are sustained. Assignment of A_undiff_ to these distinct states would affect the balance between self-renewal and differentiation plus functional activity of the A_undiff_ compartment. Replenishment of cells in the differentiation-primed state and restoration of self-renewing fractions after genotoxic damage is possible via dynamic interconversion of A_undiff_ states.

Our data argue against strict hierarchical organization of A_undiff_ fractions akin to that for ID4+ and ID4− A_undiff_^53^. *Id4* also appears more broadly expressed in A_undiff_ than proposed^5^, complicating interpretation of this model. Oct4-GFP+ PDX1− EOMES− progenitor fractions readily generate Oct4-GFP− PDX1+ EOMES+ cells upon culture and Oct4-GFP+ A_undiff_ transplant and generate GFP− A_undiff_ despite minimal Oct4-GFP expression within the GFRα1+ self-renewing pool. Similarly, PDX1− A_undiff_ generate transplantation colonies containing PDX1+ cells. Our data is consistent with a dynamic stem cell compartment in which A_undiff_ exhibit context-dependent stem cell activity^4^. The frequency of cellular transitions under physiological conditions needs to be considered. Oct4-GFP marks GFRα1− SOX3+ RARγ+ A_al_, cells that co-express *Ngn3* and are differentiation-destined in undisturbed testis^19,21^. This fate is dictated by RA sensitivity and periodic RA pulses^11^. Differentiation commitment is also associated with A_undiff_ eviction from the niche^39^. Placing differentiation-primed A_undiff_ in an in vitro environment lacking exogenous RA and containing supraphysiological levels of niche factors can be predicted to restore a self-renewing state. Endogenous stem cell depletion in transplantation recipients permits access of differentiation-primed cells to the niche and an environment promoting self-renewal. Such settings are however unable to rescue self-renewal capacity once A_undiff_ differentiate^54^. Our observations highlight a limitation of the transplant assay; A_undiff_ destined to differentiate in undisturbed tissue still generate colonies in a transplant setting^9,13^. Lineage tracing provides better insight into cell fate in vivo but also suffers limitations^55^.

Interconversion of distinct states within the GFRα1+ pool evidently occurs in vivo. Formation of PDX1+ A_undiff_, presumably from GFRα1+ PDX1− cells, is observed during postnatal development. Transplanted PDX1− A_undiff_ generate PDX1+ cells, mimicking this developmental transition. Upon germline recovery following busulfan, the PDX1+ population is restored, potentially from GFRα1+ EOMES+ cells. GFRα1+ cells may routinely transit through distinct states even in steady state while Oct4-GFP+ progenitors must be removed from their physiological context to revert efficiently to a self-renewing state. Lineage tracing studies, e.g., of the GFRα1+ PDX1− population, will be needed to confirm cellular transitions in situ.

Comparison of the male germline with other high-turnover tissues, e.g., haematopoietic, can provide insight into stem cell characteristics in developing and homeostatic settings. Haematopoietic stem cells (HSCs) of the foetal mouse are more mitotically active than adult counterparts and have higher self-renewal capacity^56^. A switch in murine HSC activity occurs 3–4 weeks postnatally, accompanied by changes in gene expression and differentiation potential^57^. Self-renewal mechanisms of foetal and adult HSCs are distinct and postnatal transition in HSC properties regulated through intrinsic cues^58,59^. While transplantable stem cells are present in early postnatal testis onwards, age-dependent changes in A_undiff_ properties and self-renewal mechanisms are poorly appreciated. Our data indicate that common to HSCs, A_undiff_ are more mitotically active in neonates than adults. Transplantation studies demonstrated that stem cells from pups have altered proliferative capacities and differentiation tendencies than those of adults^51^. *Pdx1* is induced in a population of A_s_ and A_pr_ in young adults that possess potent transplantation capabilities, suggesting that PDX1 marks the onset of a mature stem cell phenotype. After germ cell depletion by busulfan, *Pdx1* is downregulated in regenerating A_undiff_ and PDX1+ populations only recover upon epithelium renewal. Similar demands are likely placed on stem cells in developing and regenerating tissues, i.e., founder/remaining stem cells expand rapidly to establish/restore the stem cell compartment then generate differentiating cells. Loss of PDX1+ A_undiff_ during regeneration is consistent with development of this population in homeostatic testis. It will be of great interest to characterize phenotypic and functional differences between A_undiff_ under homeostatic vs. developmental or regenerative conditions and factors responsible for divergent properties.

Given that PDX1+ A_undiff_ development coincides with maturation of supporting Sertoli cells and GDNF promotes adoption of a PDX1+ cell state in vitro^60^, extrinsic cues can drive formation of this population. Genes sharing similarly restricted expression as *Pdx1* in adult A_undiff_ (*Lhx1*, *Eomes*, *T*) are positively regulated by GDNF in cultured A_undiff_, supportive of a role for this niche factor^16,33^. However, GDNF levels peak in early postnatal testis and are induced following busulfan-mediated germ cell depletion^14,61^, conditions in which PDX1+ cells are not found. The presence of high bFGF levels in addition to GDNF may promote A_undiff_ expansion under these conditions but prevent adoption of the PDX1+ state^62^. EOMES+ A_undiff_ are present during postnatal development and enriched in regenerative testis, consistent with GDNF-dependent regulation. Whether conserved signals regulate *Pdx1* expression in foregut tissues and postnatal testis remains to be determined. Selective induction of genes such as *Pdx1* in adult stem cells indicates that besides neonatal gonocyte-to-spermatogonia transition, stem cell formation involves an additional developmental step occurring upon exposure of nascent A_undiff_ to a mature testis environment.

## Methods

### Mouse generation and manipulation

The Plzf-mC/CreER transgenic reporter line was generated at the Monash Gene Targeting Facility through standard pro-nuclear injection techniques. Recombineering was used to construct the transgene from a BAC template. The mCherry-T2A-CreERT2 cassette replaces the second exon after the translation initiation site and is followed by a poly(A) sequence. The transgene incorporates 20 kb upstream *Plzf*, the first untranslated exon and first intron plus 1 kb of the second intron. Two independent founder lines were established and mice from the first line were used for the majority of experiments. Z/EG, *Sox2*^GFP^, Oct4-GFP, *Pdx1*^GFP^ and *Id4*^IRES-GFP^ lines have been described elsewhere^25,28,32,38,40^. Hemizygous or heterozygous reporter mice were generally maintained on a mixed C57BL6/CBA background except Oct4-GFP (C57BL6/CBA/129T2svJ) and *Id4*^IRES-GFP^ (FVBN/CBA) lines. For lineage tracing, Plzf-mC/CreER; Z/EG mice (4–6 weeks old) were injected intraperitoneally daily with 2 mg tamoxifen (Sigma-Aldrich) in sesame oil for 5 consecutive days^22^. Wild-type mice for analysis of testis development and regeneration were C57BL6. Adult mice were generally harvested at 8–10 weeks of age. Busulfan (Cayman Chemical) was prepared for intraperitoneal injection as described^22^. Spermatogonial transplantation was performed using busulfan-conditioned C57BL6/CBA F1 recipients^20^. A volume of 10–15 μl of donor cell suspension was microinjected via the testis efferent ducts. Recipients of cells from Oct4-GFP mice were treated with neutralising antibody to CD4 to promote immune tolerance^35,63^. All animal experiments were subject to approval by the Monash University Animal Ethics Committee.

### Immunofluorescence

For frozen sections, testes were harvested and fixed in 4% paraformaldehyde (PFA) overnight at 4 °C then washed in phosphate-buffered saline (PBS) prior to cryoprotection with 30% sucrose in PBS and embedding in OCT. Sections (8 μm) were processed as previously detailed^20^ and blocked with 10% foetal bovine serum (FBS) or donkey serum with 2% bovine serum albumin (BSA) in PBS. Whole-mount immunostaining of seminiferous tubules is described elsewhere^48^. For immunostaining of cultured A_undiff_, cells were plated onto Lab-Tek II chamber slides (Thermo Fisher Scientific) coated with Geltrex (Thermo Fisher Scientific) and allowed to form colonies. Cells were fixed in 4% PFA for 10 min at room temperature and washed in PBS prior to permeabilization for 10–15 min in methanol at −20 °C. Slides were blocked and stained as for sections above. Primary antibodies were as follows: Goat anti-GFRα1 (AF560, 1:250), anti-c-KIT (AF1356, 1:250), anti-mouse/rat PDX1 (AF2517, 1:250), anti-PLZF (AF2944, 1:500), anti-LIN28A (AF3757, 1:500) and anti-SOX3 (AF2569, 1:250) (R&D Systems), rabbit anti-mCherry (ab167453, 1:2000), anti-OCT4 (ab19857, 1:500), anti-SALL4 (ab29112, 1:2000) (Abcam), chicken anti-GFP (ab13970, 1:5000) and rat anti-germ cell-specific antigen (TRA98, 1:500) (Abcam), rat monoclonal anti-mCherry clone 16D7 (Thermo Fisher Scientific, 1:2000), rabbit monoclonal anti-Cyclin D1 clone SP4 (1:250) and mouse monoclonal anti-DNMT3A clone 64B1446 (1:200) (Novus Biologicals), rat anti-KI67 clone SolA15 (eBioscience, 1:250), rabbit monoclonal anti-c-KIT clone D13A2 (1:400) and anti-RARγ clone D3A4 (1:500) (Cell Signaling Technology) and rabbit monoclonal anti-mouse EOMES clone 1219A (R&D Systems, 1:1000). Primary antibodies were detected with appropriate Alexa Fluor-conjugated secondary antibodies (Thermo Fisher Scientific and Jackson ImmunoResearch, 1:500) and samples counterstained with DAPI prior to mounting with Vectashield mounting medium (Vector Labs). Samples were analysed on a Zeiss LSM 780 confocal microscope (Monash Micro Imaging).

### Flow cytometry

To generate single-cell suspensions from adult testis, dissected seminiferous tubules were coarsely minced and washed with PBS then digested with 1 mg/ml collagenase Type IV (Sigma-Aldrich) in unsupplemented DMEM medium and 50 μg/ml DNase I. Tubule fragments were washed in PBS to deplete interstitial cells and peritubular myoid cells then dissociated with 0.25% trypsin in the presence of DNase I and passed through a 70 μm cell strainer. For live analysis and sorting, cells were stained with FITC (1:250) or biotin-conjugated (1:500) anti-CD9 clones KMC8 or MZ3 (eBioscience and Biolegend) and allophycocyanin (APC)-conjugated anti-c-KIT clone 2B8 (eBioscience, 1:500). Biotinylated antibody was detected with streptavidin conjugated to APC-eFluor 780 (eBioscience, 1:2000) and DAPI used for live/dead discrimination. Spermatogonia-enriched fractions for sorting were prepared by CD9 selection using an EasySep biotin selection kit (Stem Cell Technologies) and biotinylated CD9 antibody (Biolegend clone MZ3, 1:500) according to manufacturers instructions. For intracellular staining, testis cell suspensions were fixed in PFA then permeabilized in methanol on ice for 30 min or overnight at −20 °C. Fixed and permeabilized cells were washed in PBS with 2% FBS then stained with the following antibodies: chicken anti-GFP (Abcam ab13970, 1:5000), eFluor 450-conjugated anti-Ki67 clone SolA15 (1:500) and phycoerythrin (PE)-conjugated anti-c-KIT clone 2B8 (1:250) (eBioscience), rabbit polyclonal anti-mCherry (Abcam ab167453, 1:1000), goat anti-mouse/rat PDX1 (R&D Systems, AF2517, 1:300), rabbit anti-mouse EOMES clone 1219A (R&D Systems, 1:1000) and Alexa 647-conjugated anti-PLZF clone 9E12 (1:2000)^20^. Antibodies to EOMES, GFP, mCherry and PDX1 were detected with Alexa 488 and 568 conjugated secondary antibodies (Thermo Fisher Scientific and Jackson ImmunoResearch, 1:500). Cells were analysed on an LSR Fortessa and sorted with a BD Influx Cell Sorter (BD Biosciences). Data were processed with FlowJo software.

### Cell culture

A_undiff_ cultures were established and maintained on mitomycin-inactivated mouse embryonic fibroblast (MEF) feeders in StemPro-34 media (Thermo Fisher Scientific) supplemented with 10 ng/ml GDNF, 10 ng/ml bFGF, 20 ng/ml EGF, 25 μg/ml insulin and other additives as described^20,64^. Cultures between passages (P) 3 and 8 were used unless indicated otherwise and harvested using Trypsin/EDTA. For analysis of cell growth and GFP expression by flow cytometry, cells were plated onto 24-well plates at indicated densities and followed over time. When analysed by flow cytometry, A_undiff_ were distinguished from contaminating feeders by mCherry expression. For IF, cells were plated onto four-well chamber slides coated with Geltrex (Thermo) as detailed^22^. GDNF levels in conditioned media from cells cultured on 12-well plates were measured with a DuoSet ELISA system (R&D Systems). To induce differentiation, cells were treated with 1 μm retinoic acid in DMSO (Sigma-Aldrich) for 48 h. For transplantation, cells were dissociated with Trypsin/EDTA and resuspended at 5 × 10^6^ cells/ml in germ cell media supplemented with 50 μg/ml DNase I and 10% Trypan blue (Sigma-Aldrich). Inhibitors to MAPK (PD0325901; 5 μm), PI3Kα (BYL719; 1 μm) and mTOR (Torin; 0.5 μm) (Selleckchem) were dissolved in DMSO then diluted in media. Inhibitor-containing media was replaced each day for 4 days prior to cell analysis.

### Microarray and qRT-PCR

Sorted cells were lysed in TRIzol LS reagent (Thermo Fisher Scientific) then RNA was purified and DNase treated using a Direct-zol RNA Miniprep kit (Zymo Research). Gene expression in A_undiff_ fractions from pooled Oct4-GFP; Plzf-mC/CreER mice (three independent sorts) was analysed with an Agilent Technologies SurePrint G3 8 × 60 K microarray at the Monash Health Translation Precinct Genomics Facility. Significance was determined by paired *t* test (*P* < 0.05) with 1.5-fold change cutoff. For qRT-PCR, cDNA was synthesized from isolated RNA using a Tetro cDNA synthesis kit (Bioline). Quantitative PCRs were run on a Roche LightCycler 480 using Takara Sybr Premix Ex Taq II (Clontech). Primer sequences were as previously described^20,23^ or obtained from the Harvard PCR Primer Bank (https://pga.mgh.harvard.edu/primerbank/) and listed in Supplementary Table 1.

### RNA-sequencing

RNA was purified from sorted cells and DNase treated using a Direct-zol RNA Microprep kit (Zymo Research). RNA quality and quantity was assessed using a Bioanalyser and Qubit. SPIA amplification and cDNA generation was performed using 2 ng total RNA as per Nugen Ovation RNA-seq system V2 protocol followed by Ovation Ultralow System V2 for library preparation. SPIA amplified cDNA was quantitated and 100 ng sheared using Covaris sonication and processed. Seven cycles of amplification were used to minimize any amplification effects. Library size (~320 bp) was checked by Bioanalyzer. Libraries were quantified by Qubit and qPCR and one equimolar pool made based upon qPCR results. Following denaturation, 200 pM of library pool was clustered in one lane of a HiSeq 3000 8-lane flowcell using c-Bot. RNA-sequencing was performed at the Medical Genomics Facility, Monash Health Translation Precinct (MHTP). Data was processed by the Monash University Bioinformatics Platform using the RNAsik pipeline. Raw reads (FASTQ) were mapped against Ensembl reference files (FASTA and GTF) followed by read counting. Differential gene expression was performed with Degust webtool, based around the R package *limma* using limma-voom for statistical analysis. Differentially expressed genes were classified using the DAVID Bioinformatics Resource and KEGG pathway database^65^.

### Single-cell PCR

Preamplified template for single cell PCR was generated with Fluidigm’s C1 platform as per instrument specifications. In two independent experiments a total of 152 mCherry+ CD9+ c-KIT− cells isolated from pooled Plzf-mC/CreER adult testis were captured and preamplified with 71 TaqMan probes to the following genes (those identified as differentially expressed in Oct4-GFP+ and GFP− A_undiff_ fractions by microarray are underlined): *Actb*, *Bcl6b*, *Ccnd2*, *Ccne1*, *Cd82*, *Cxcr4*, *Ddit4*, *Ddx4*, *Dmrt1*, *Dnmt3a*, *Dnmt3b*, *Dnmt3l*, *Dppa2*, *Dppa3*, *Egr2*, *Eif4b*, *Elk3*, *Eomes*, *Esr1*, *Etv1*, *Etv4*, *Etv5*, *Fbxw7*, *Fgfr1*, *Fgfr2*, *Foxo6*, *Gfi1*, *Gfra1*, *Hprt*, *Id4*, *Ifitm1*, *Ifitm3*, *Itga6*, *Itgb1*, *Kit*, *Lhx1*, *Lhx2*, *Lin28a*, *Myc*, *Nanos2*, *Nanos3*, *Neurod1*, *Ngn3*, *Notch1*, *Pax7*, *Pdx1*, *Pou3f1*, *Pou3f2*, *Pou5f1*, *Rarg*, *Ret*, *Rpl32*, *Rspo1*, *Sall1*, *Sall4*, *Six2*, *Smad6*, *Snai1*, *Sohlh1*, *Sox2*, *Sox3*, *Sox10*, *Stra8*, *T*, *Tbx20*, *Tcl1*, *Tet3*, *Thy1*, *Upp1*, *Zbtb16* and *Zic2*. Single-cell PCR data collection was performed with a Biomark instrument (Fluidgm). Results are expressed as Log2Ex = LOD (Limit of Detection) Cq − Cq [Gene]. The limit of detection was set to 28. If Log2Ex value is negative, Log2Ex = 0. Summary statistics and plots were performed and produced using made4, caroline and gplots^66,67^. viSNE, a visualization tool for high-dimensional single-cell data based on the Barnes-Hut implementation of the t-Distributed Stochastic Neighbour Embedding (t-SNE) algorithm was applied to the data set^34^. In detail, 0.1 was added to Log2Ex values and resulting tables converted to flow cytometry standard files. The Cytobank platform (Fluidgm, South San Francisco, California) was used for generation of viSNE maps.

### Single-cell RNA-sequencing

mCherry+ CD9+ c-KIT− A_undiff_ fractions were independently isolated from 2 Plzf-mC/CreER; Oct4-GFP adults and resuspended at ~300 viable cells/μl in PBS containing 0.04% BSA. Library construction was performed using a 10 × Chromium controller with the Chromium Single Cell 3′ Reagent Kit V2. Both libraries were sequenced in one high-output lane of an Illumina NextSeq in single-read 150b format (115b effective read length). Single-cell (sc) RNA-seq processing and analysis was performed with Cell Ranger software (10× Genomics, Inc.) and Monocle^36,68,69^. scRNA-seq samples were demultiplexed with cellranger mkfastq. Sequencing reads were aligned to a custom mouse genome (Cell Ranger mm10 version 1.2.0, including GFP and mCherry sequences as extra chromosomes). The annotation file was modified to count reads assigned to both GFP and mCherry cassettes and UMIs counted with cellranger count. Both libraries were aggregated and normalized by depth, to have the same of number of mapped reads, with cellranger aggr. Gene expression matrix, containing both cell and expression details, were processed for downstream analysis using both cellRanger’s load_cellranger_matrix and Monocle’s newCellDataSet function (lowerDetectioLimit = 0.5 and expressionFamily = negbinomial.size()).

Analysis was performed using components of Monocle’s package unless otherwise stated. Cell counts were normalised with the estimateSizeFactors function previously discarding mitochondrial and ribosomal associated genes^70^. Cell gene expression was detected using detectGenes (min_expr = 0.1) and genes not expressed in at least 175 cells were discarded. Dispersion was estimated for the remaining gene set using estimateDispersions^70^. Cells with a total number of mRNA (log10) within two standard deviations from the mean were kept for further analysis.

In a first instance, cell classification was performed with classifyCells based on the following criteria: contaminant cells were defined as those cells that expressed at least one transcript of *Acta2*, *Icam2* or *Cdh5* marker genes; germ cells were those cells that expressed at least one transcript of *Zbtb16*, *Ddx4* or *Sall4* and did not express any of the contaminant cell marker genes. Only those cells classified as germ cell were used for downstream analyses. Cells with no detectable expression of the housekeeping gene *Gapdh* (senescent) were also excluded from the analysis.

For single-cell trajectory analysis, germ cells were ordered in a semi-supervised mode using a cell type classification based on the following criteria: transitional cells were defined as those cells that express at least one transcript of both stem (*Etv5* and *Gfra1*) and progenitor (*Upp1* and *Sox3*) marker genes; stem cells were defined as those cells that expressed at least one transcript of the stem cell marker genes but did not express progenitor marker genes; progenitor cells were defined as cells that expressed at least one transcript of the defined progenitor marker genes and did not express stem cell marker genes. A minor fraction of cells was not classified as either stem or progenitor cells based on the described markers and were referred to as undefined undifferentiated. The makerDiffTable function (residualModelFormulaStr = “~Library+ num_genes_expressed”) was used to identify those genes that were co-expressed with markers that define cell type hierarchy described above (top 2500 genes based on q value were selected as ordering genes). Dimensionality reduction was performed with reduceDimension using the discriminative dimensionality reduction with trees (DDRTree) method and log normalization. Cells were ordered along the trajectory with orderCells (reverse = T). Differential gene expression test along the cell trajectory was performed with differentialGeneTest (fullModelFormulaStr = “~sm.ns(Pseudotime)+ Library+ num_genes_expressed” and reducedModelFormulaStr = “~Library+ num_genes_expressed”). Cell trajectory, gene expression across pseudotime and heatmap of pseudotime plots were created with plot_cell_trajectory, plot_genes_in_pseudotime (min_expr = 0.1, trend_formula = “~ sm.ns(Pseudotime, df = 4)”) and plot_pseuodtime_heatmap respectively.

### Statistical analysis

Assessment of statistical significance was performed using a two-tailed unpaired *t*-test (GraphPad Prism). *P*-values are indicated as follows: *P < 0.05; **P < 0.01; ***P < 0.001; ****P < 0.0001; not significant (ns) *P* > 0.05. For mouse experiments, no statistical method was used to predetermine sample sizes and no specific randomization or blinding methods were used.

### Data availability

Microarray and RNA-Seq data are deposited in the Gene Expression Omnibus (GEO) database as a SuperSeries with accession number GSE107256. All other relevant data are available from the corresponding author on request.

## Electronic supplementary material

Supplementary Information

Description of Additional Supplementary Files

Supplementary Data 1

Supplementary Data 2
