# Supplementary material for: Identification of dynamic undifferentiated cell states within the male germline
Source: Nat Commun. 2018 Jul 19;9:2819. doi: 10.1038/s41467-018-04827-z (PMC6053434; doi:10.1038/s41467-018-04827-z)
Supplement: Supplementary file 1 — Supplementary Information [file 41467_2018_4827_MOESM1_ESM.pdf]

SUPPLEMENTARY INFORMATION

Identification of dynamic undifferentiated cell states within the male germline

La, Mäkelä et al.

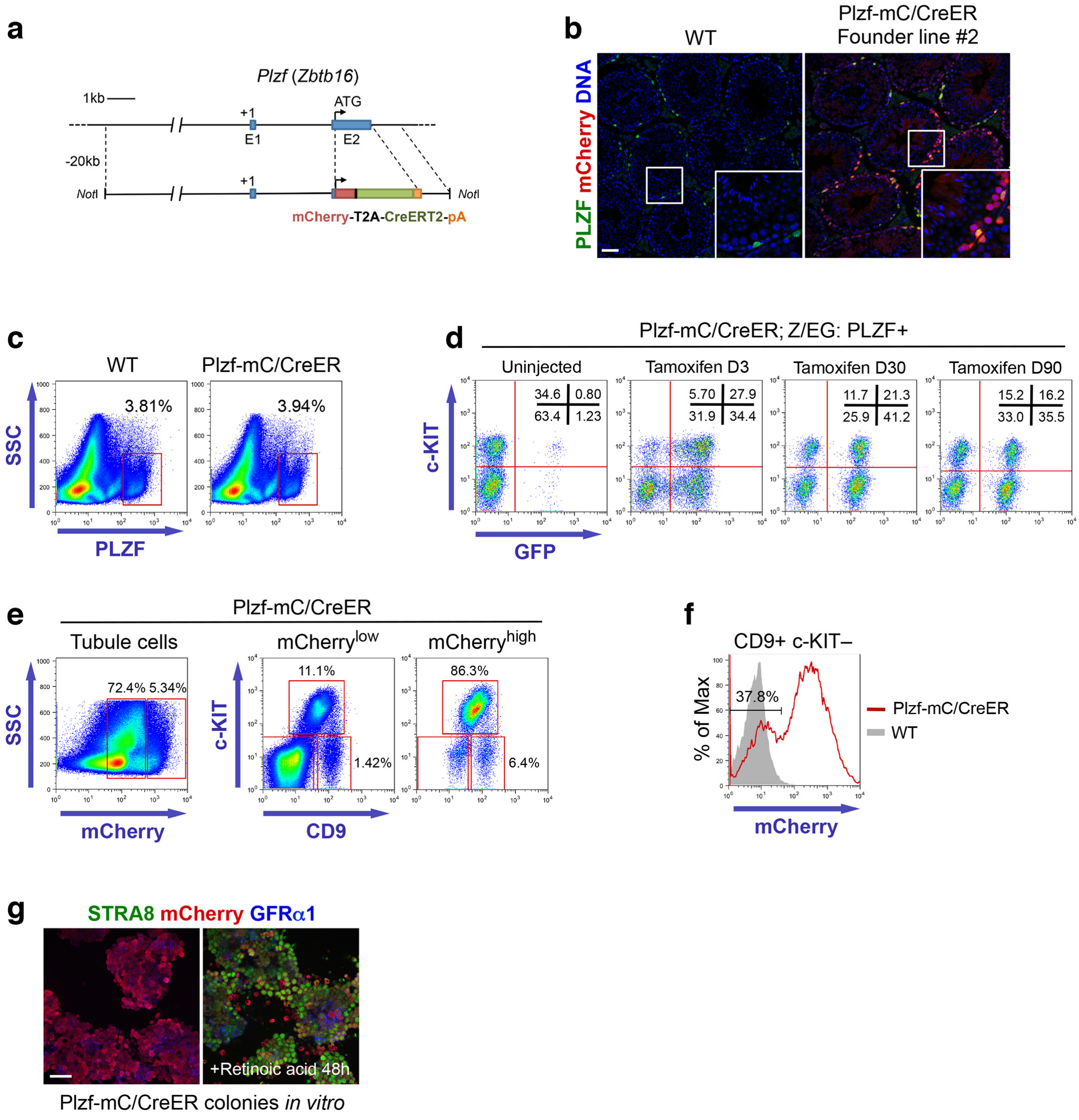

Supplementary Figure 1. Characterizing Plzf-mC/CreER transgenic mice and cultured A_{undiff} cells. (**a**) Schematic of Plzf-mC/CreER transgene and comparison to upstream region and first 2 exons of *Plzf* (7 exons total). Indicated regulatory elements of *Plzf* drive expression of a mCherry-T2A-CreERT2 cassette. A poly(A) (pA) sequence is included at the cassette 3' end. Exons (E) of *Plzf* are shown and homologous regions of gene and transgene are outlined with dashed lines. Transcription start site is indicated as +1 and translation start site as arrowhead and ATG. (**b**) Representative immunofluorescence (IF) of adult testis sections from Plzf-mC/CreER mice of founder line #2 and wildtype (WT) controls. DNA is counterstained with DAPI. Insets show higher magnification detail of indicated area. Scale bar, 50 µm. (**c**) Flow cytometry analysis of fixed and permeabilized testis cells from Plzf-mC/CreER and WT control adults. PLZF+ gating strategy was set according to isotype-stained control. Percentage of cells contained within respective gates is indicated (n=3 mice per genotype). (**d**) Plzf-mC/CreER; Z/EG mice were injected daily with tamoxifen for 5 days then harvested at the indicated days (D) after last treatment. Fixed and permeabilized testis cells were analyzed by flow cytometry. Representative profiles of PLZF+ cell populations are shown. Percentage of cells within respective gates is indicated (n=4 testes D3 and D90, n=6 testes D30). (**e**) Representative flow cytometry analysis of live testis cells from adult Plzf-mC/CreER mice. mCherry+ cells were divided into 2 fractions according to level of mCherry expression. Gates were set according

to wildtype isotype-stained controls and percentage of cells within gates is shown. SSC is side scatter. (**f**) Histogram shows mCherry expression within CD9+ c-KIT– fraction of adult Plzf-mC/CreER testis. Representative profile from 1 of 3 mice is shown and fraction of mCherry– cells indicated. Gray filled profile is equivalent fraction from WT control. (**g**) Representative IF of colonies from cultures of sorted adult Plzf-mC/CreER A_{undiff} treated with vehicle or retinoic acid for 48 hours (n=3 cultures). Scale bar, 50 μm.

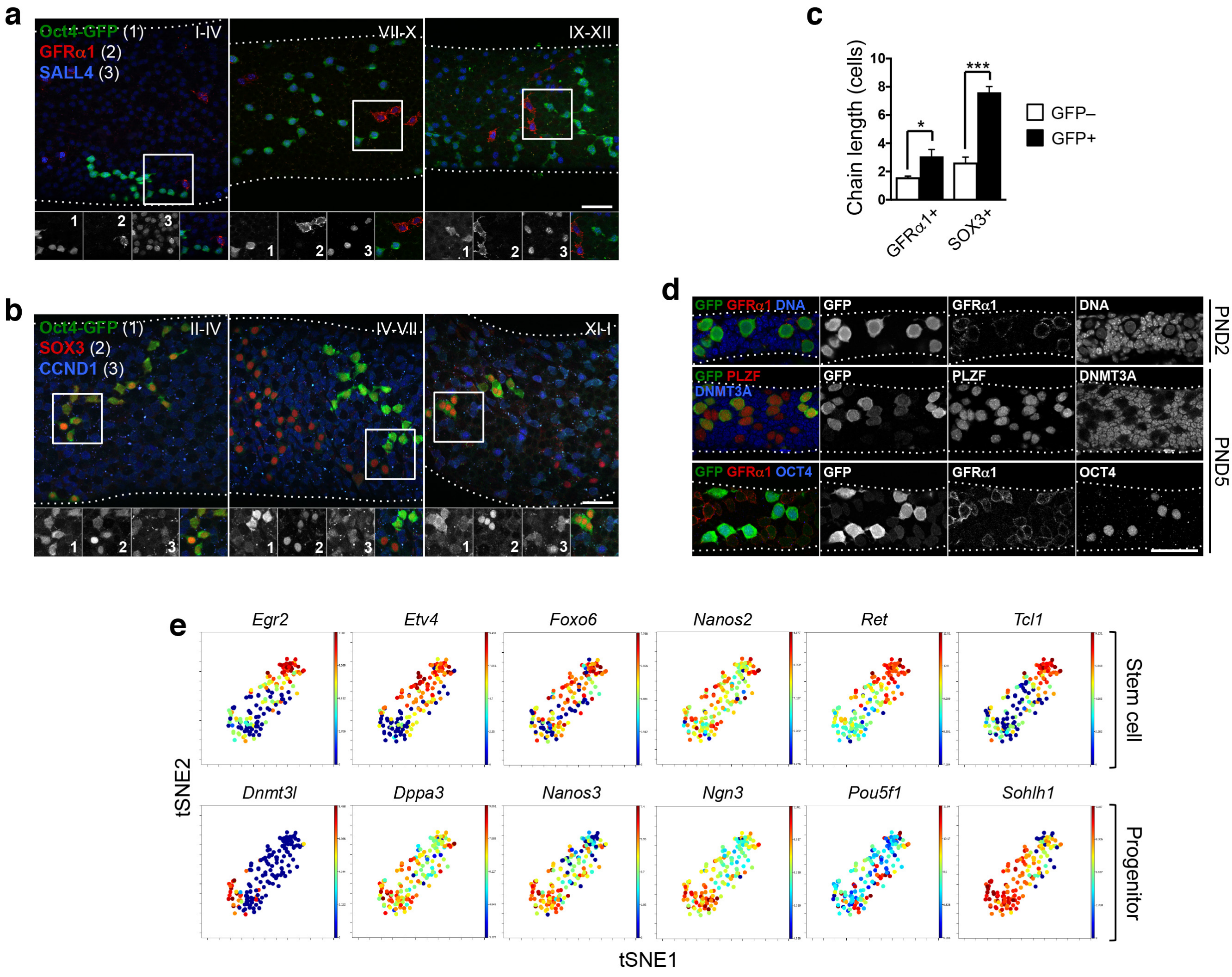

Supplementary Figure 2. Expression pattern of *Oct4* in the male germline. (**a**, **b**) Representative wholemount IF of adult Oct4-GFP seminiferous tubules at different stages (n=3 mice). Grayscale panels show individual immunostaining within indicated area at higher magnification. Roman numerals indicate cycle stage of the seminiferous epithelium. Spermatogonia were counterstained for SALL4 in **a**. Cyclin D1 (CCND1) marks differentiating spermatogonia in **b**. Scale bars, 50 μm. (**c**) Graph indicates length of GFP– and GFP+ spermatogonial chains within the GFRα1+ and SOX3+ positive populations from wholemount analysis of adult Oct4-GFP seminiferous tubules. Spermatogonial identity was confirmed by SALL4 counterstain. Mean values ± s.e.m are shown (n=3 mice, >200 cells scored per population). Significance was calculated by two-tailed Student's *t*-test (*P < 0.05, ***P < 0.001). (**d**) Representative whole-mount IF staining of seminiferous tubules from Oct4-GFP neonates of the indicated ages (PND; postnatal day). n=3 mice of each age. Scale bar, 50 μm. (**e**) viSNE maps derived from single cell analysis of sorted A_{undiff} from Plzf-mC/CreER adults. Based on expression of a set of 71 candidate genes in 150 cells. Each plot point represents one cell and proximity of cell points reflects combined similarity in gene expression. Relative expression of genes associated with stem and progenitor fractions by each cell is indicated (red=high, blue=low). *Pou5f1* (*Oct4*) is in lower row.

La, Mäkelä et al. Supplementary Figure 3

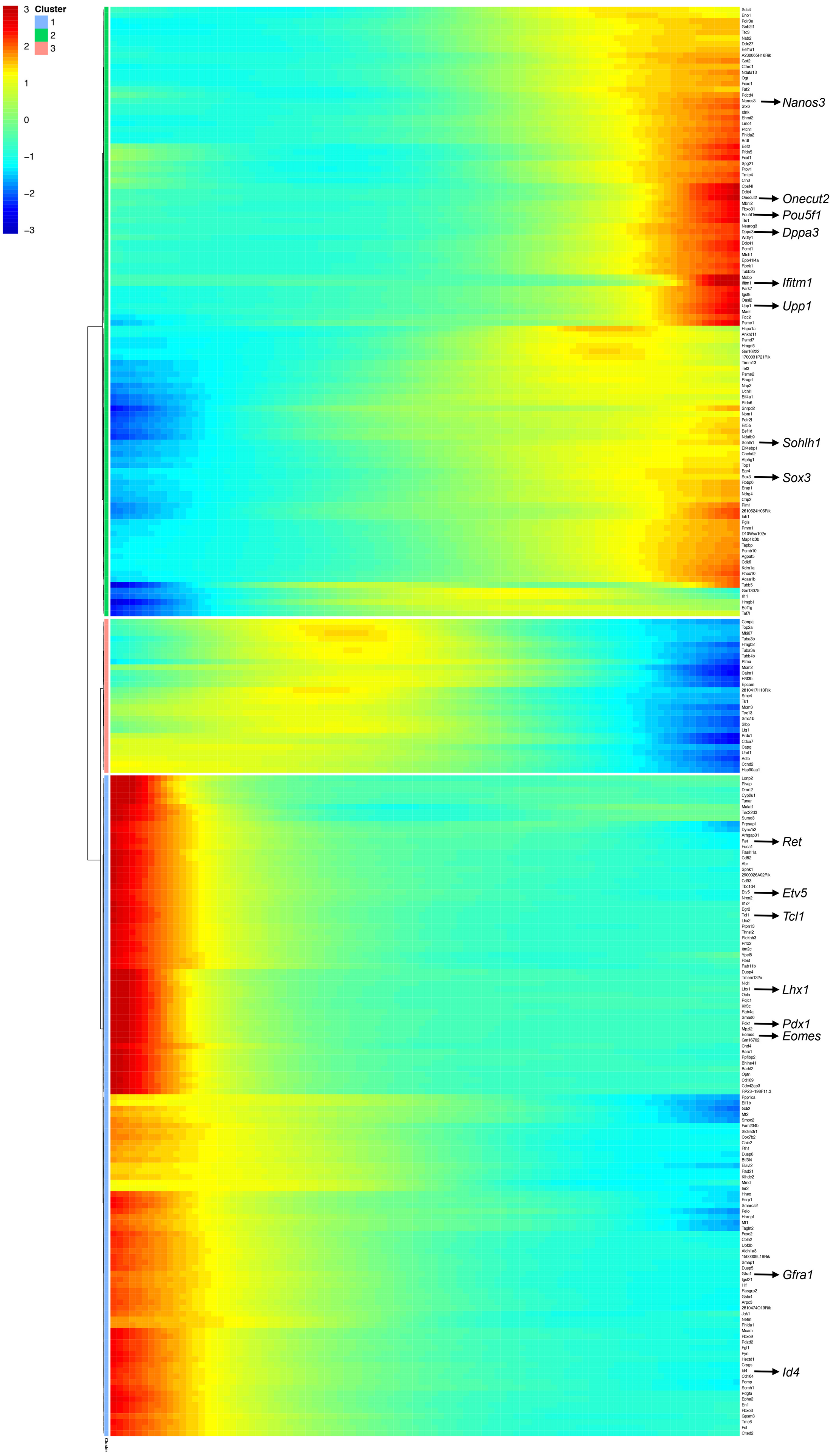

Supplementary Figure 3. Differentially expressed genes (DEG) during A_{undiff} differentiation priming. Isolated A_{undiff} from Plzf-mC/CreER; Oct4-GFP adults were analysed by single cell RNA-Seq and developmental trajectory (pseudotime) of ~3500 germ cells calculated using Monocle in semi-supervised mode. Heatmap shows top 250 DEG (based on q values) across the cell trajectory (correlating to differentiation priming) with genes of interested highlighted. The following 3 gene clusters showing related expression patterns were identified: #1 Genes downregulated across pseudotime (stem cell associated); #2 Genes upregulated across pseudotime (progenitor-associated); #3 Genes transiently upregulated through pseudotime (associated with stem to progenitor transition).

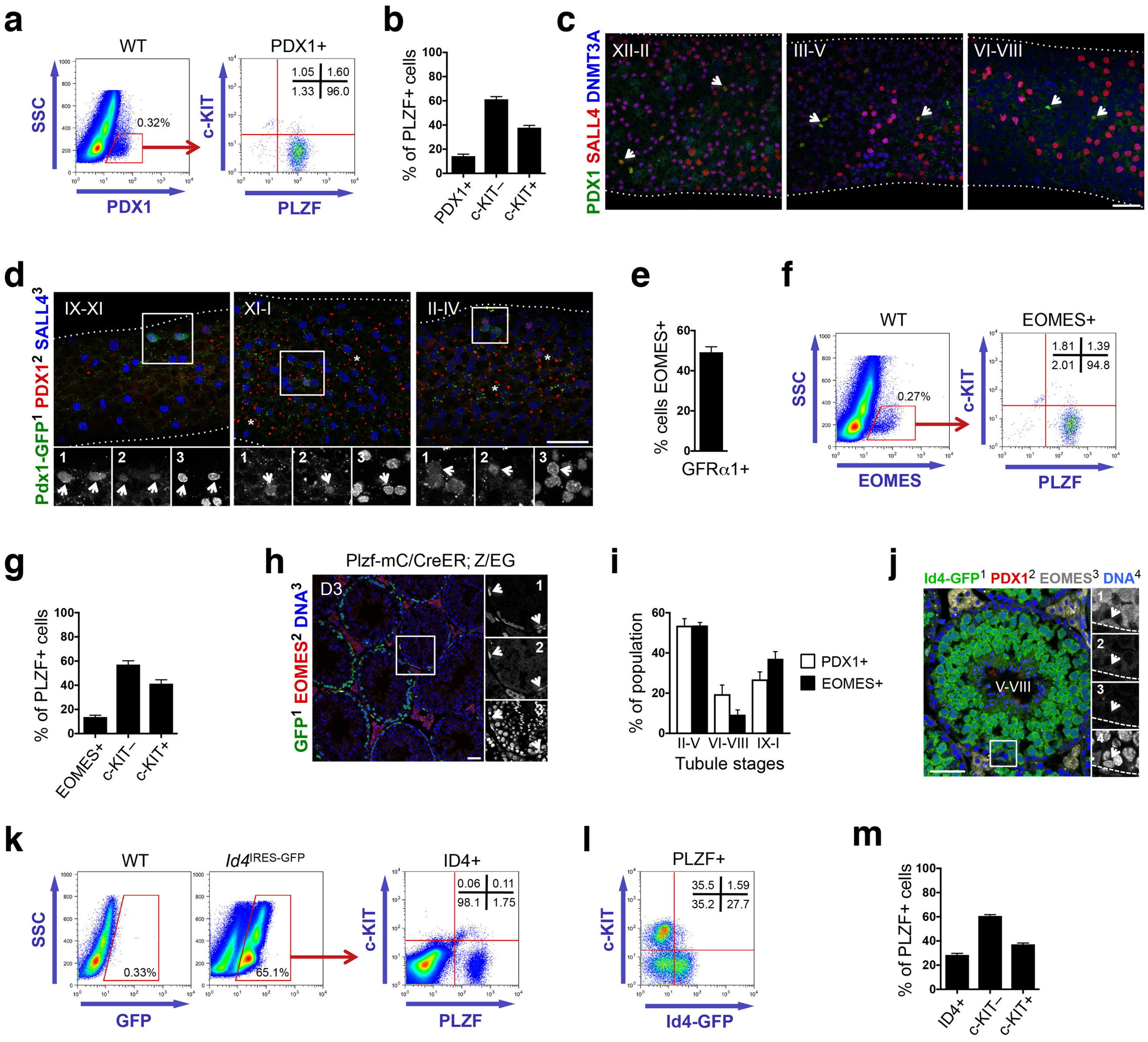

Supplementary Figure 4. Characterization of A_{undiff} populations in adult testis. (**a**) Flow cytometry of fixed and permeabilized testis cells from wildtype (WT) adult for PDX1. Percentage of cells in each gate from representative sample is indicated. Gates were set according to isotype and secondary antibody-stained controls. (**b**) Quantification of flow cytometry from analysis in **a**. Bar chart shows relative abundance of the PLZF+ fractions. Mean values are shown ± s.e.m. (n=3 mice). Note that PDX1+ cells are a minor subset of the PLZF+ population. The majority of PLZF+ cells are c-KIT– (A_{undiff}) with a smaller although significant c-KIT+ differentiating fraction. (**c**) Representative wholemount IF of adult WT seminiferous tubules at different stages (indicated by Roman numerals) (n=3 mice). Scale bar, 50 µm. (**d**) Representative wholemount IF of adult *Pdx1*^{GFP} seminiferous tubules showing overlap of GFP and *Pdx1* expression at different stages (arrowheads). Grayscale panels show details of individual immunostaining of indicated areas. Staging is indicated in Roman numerals (n=2 mice). Asterisks indicate non-specific staining of spermatids with PDX1 antibody. Scale bar, 50 µm. (**e**) Graph shows percent GFRα1+ spermatogonia that are EOMES+ from IF of adult WT sections. Mean value ± s.e.m is shown (n=4 mice). (**f**) Flow cytometry of fixed and permeabilized testis cells from WT adult for EOMES. Percentage of cells in each gate from representative sample is indicated. Gates were set according to isotype and secondary antibody-stained controls. (**g**) Quantification of flow cytometry data from **f**. Bar chart shows relative abundance of PLZF+ fractions. Mean values are shown ± s.e.m. (n=4 mice). (**h**) Representative IF of adult Plzf-mC/CreER; Z/EG testis sections 3 days after

tamoxifen (n=2 mice). Arrowheads: lineage-marked EOMES+ spermatogonia. Inset panels show individual immunostaining within indicated area at higher magnification. Scale bar, 50 μm. (**i**) Percentage of EOMES+ and PDX1+ spermatogonia found within indicated ranges of seminiferous tubule stages from IF of WT adult testis sections. Mean values are shown ± s.e.m. (n=4 mice, between 38 and 102 cells scored per mouse). (**j**) Representative IF of testis sections from *Id4*$^{\text{IRES-GFP}}$ adults (n=4 mice). Inset panels show immunostaining within indicated area at higher magnification. Arrowhead: PDX1– EOMES– GFP+ spermatogonium. Seminiferous tubule stage is shown. Scale bar, 50 μm. (**k**,**l**) Flow cytometry of fixed and permeabilized testis cells from *Id4*$^{\text{IRES-GFP}}$ adults. Percentages of cells in each gate from representative samples are indicated (n=4 mice). Gates were set according to isotype and secondary antibody-stained WT controls. PLZF+ population is shown in **l**. (**m**) Quantification of flow cytometry from **l**. Bar chart shows relative abundance of the PLZF+ fractions. Mean values are shown ± s.e.m. (n=4 mice).

La, Mäkelä et al. Supplementary Figure 5

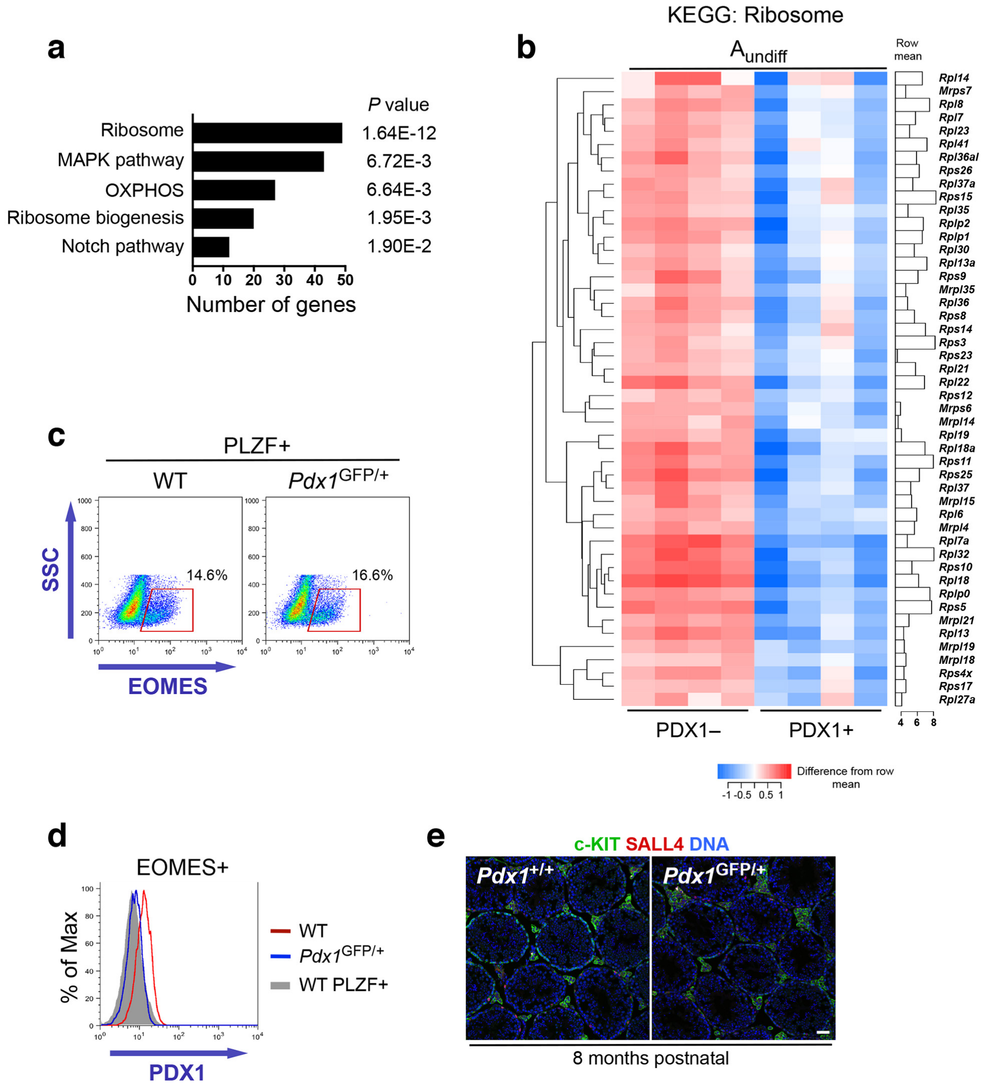

Supplementary Figure 5. Molecular characteristics of the PDX1+ A_{undiff} population. (**a**) GFP+ and GFP– A_{undiff} fractions from Plzf-mC/CreER; *Pdx1*$^{GFP/+}$ adults (n=4 mice) were analysed by RNA-Seq. Differentially expressed genes (DEG) between the populations (DEG cut-off is False discovery rate <0.05 and absolute fold change ≥1.5) were subjected to KEGG pathway analysis. Graph shows number of genes differentially expressed in a selection of identified significant pathways with associated significance values. Significance was calculated by Fisher Exact test according to the DAVID Bioinformatics Resource. (**b**) Heatmap showing differentially expressed Ribosome pathway genes from KEGG analysis of RNA-Seq data from **a**. (**c**) Representative flow cytometry analysis of fixed and permeabilized testis cells from wildtype (WT) control and *Pdx1*^{GFP} adults for EOMES (n=2 mice). SSC is side scatter. The PLZF+ cell population is shown. Percentages of cells contained within gates are indicated. (**d**) Flow cytometry analysis of PDX1 levels in EOMES+ cells from analysis in **c**. Levels in bulk WT PLZF+ fraction are included as a control (gray filled histogram). (**e**) Representative IF of testis sections from aged (8 months) mice of the indicated genotypes (n=4 mice). DNA is counterstained with DAPI. Staining for c-KIT identifies differentiating spermatogonia and early meiotic cells while SALL4 counterstain marks spermatogonia. Scale bar, 50 µm.

La, Mäkelä et al. Supplementary Figure 6

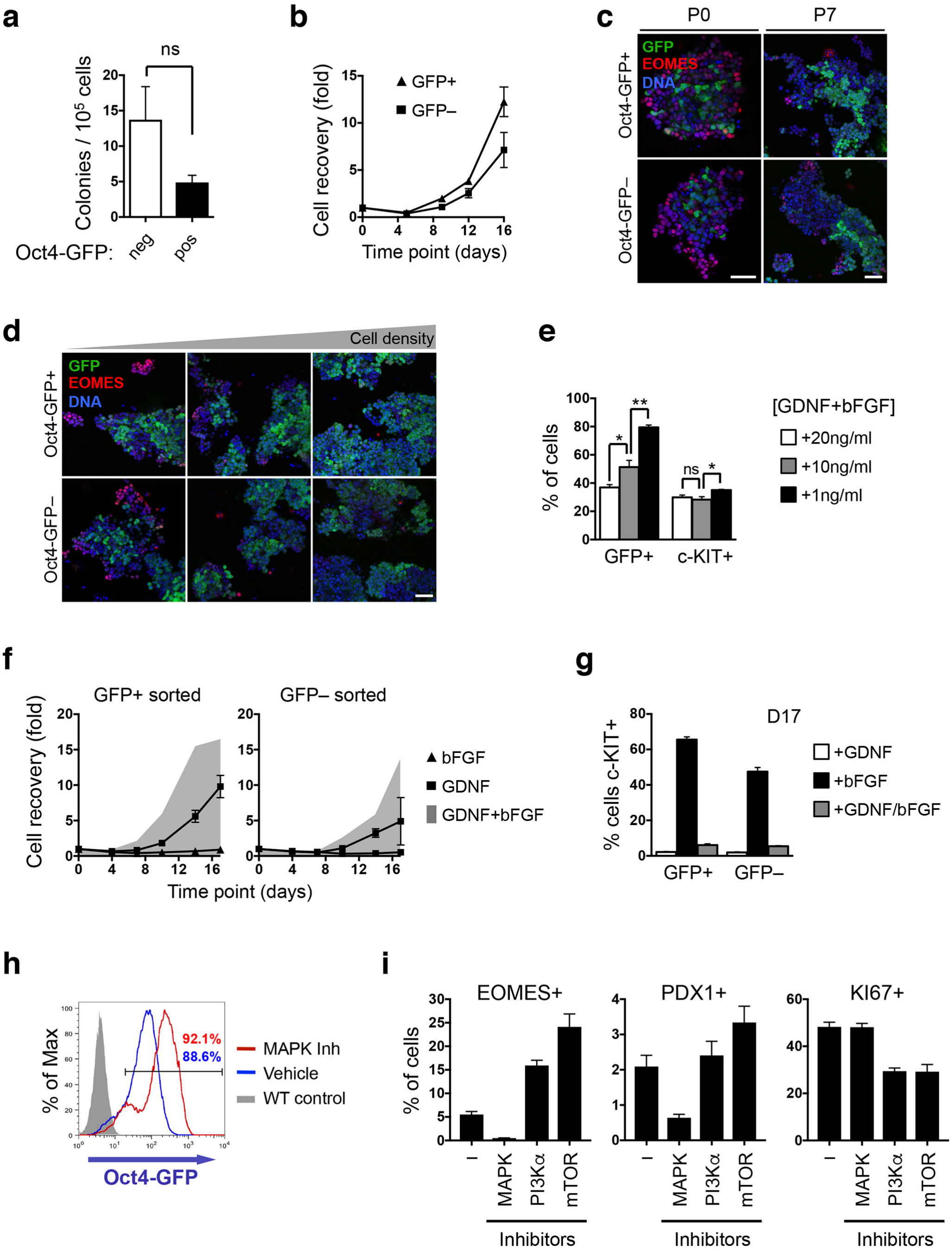

Supplementary Figure 6. Regulation of A_{undiff} heterogeneity during *in vitro* culture. (**a**) Cultures from GFP– and GFP+ A_{undiff} of Plzf-mC/CreER; Oct4-GFP adults were transplanted into busulfan-conditioned recipients. 2 sets of lines were used. Samples were harvested 8 weeks later for wholemount IF. Donor colonies were identified by mCherry IF. Mean number of colonies per 10^5 donor cells ± s.e.m. is shown (n=11 recipient testes for Oct4-GFP– cells and n=9 for Oct4-GFP+ cells). (**b**) Cultures from Oct4-GFP– and Oct4-GFP+ A_{undiff} sorted by GFP and plated at $25x10^3$/well. Cell numbers were analysed at indicated timepoints. Mean values are shown ± s.e.m. (n=6 cultures). (**c**) Representative IF of primary colonies (P0) and colonies of passage 7 (P7) cultures derived from Oct4-GFP– and GFP+ A_{undiff} (n=4 lines). Scale bar, 50 µm. (**d**) Cultured lines from Oct4-GFP– and GFP+ A_{undiff} were plated at increasing densities ($10x10^3$, $20x10^3$ and $50x10^3$ cells/well) and allowed to form colonies for 10-14 days prior to IF. Representative images are shown (n=4 lines). Scale bar, 50 µm. (**e**) Cultured cells from Oct4-GFP– A_{undiff} were plated in triplicate at $20x10^3$ cells/well. Cells were cultured for 2 weeks then switched to media containing indicated levels of GDNF and bFGF for 4 days. Mean percentages

of mCherry+ cells expressing GFP and c-KIT are shown ± s.e.m (**f**) Cultured cells from Oct4-GFP– and Oct4-GFP+ A_{undiff} sorted according to GFP and plated at $10x10^3$ cells/well in media with GDNF but no bFGF (GDNF) or bFGF without GDNF (bFGF). Cells were harvested at indicated time points. Mean fold recovery is shown ± s.d. (n=3 replicates) from a representative experiment. Gray plots indicate cells in regular media (GDNF+bFGF). (**g**) Mean percentage of cells positive for c-KIT at day 17 of experiment in **f** ± s.d. (n=3 replicates). Cells were analyzed by flow cytometry and gated according to mCherry to avoid feeder contamination. (**h**) Cultured cells from Oct4-GFP– and Oct4-GFP+ A_{undiff} were allowed to form colonies for 10-14 days prior to switching to media containing MAPK pathway inhibitor or DMSO as vehicle control. Inhibitor-containing media was replaced each day for 4 days prior to analysis by flow cytometry. mCherry+ cells were gated to exclude feeders. Histograms indicate percentage of GFP+ cells gated according to WT control. Data are from representative experiment (n=4 lines). (**i**) Cultured cells from Plzf-mC/CreER A_{undiff} were plated at $1x10^5$ cells/well, allowed to form colonies, then treated with indicated inhibitors or vehicle (–) for 4 days as in **h**. Cells were fixed and permeabilized for flow cytometry. Graphs show mean percentage of cells positive for indicated markers ± s.d. (n=3 wells per condition) from representative experiment (n=2 cultures). Significance calculated by two-tailed Student's *t*-test (*P < 0.05, **P < 0.01, not significant (ns) P > 0.05).

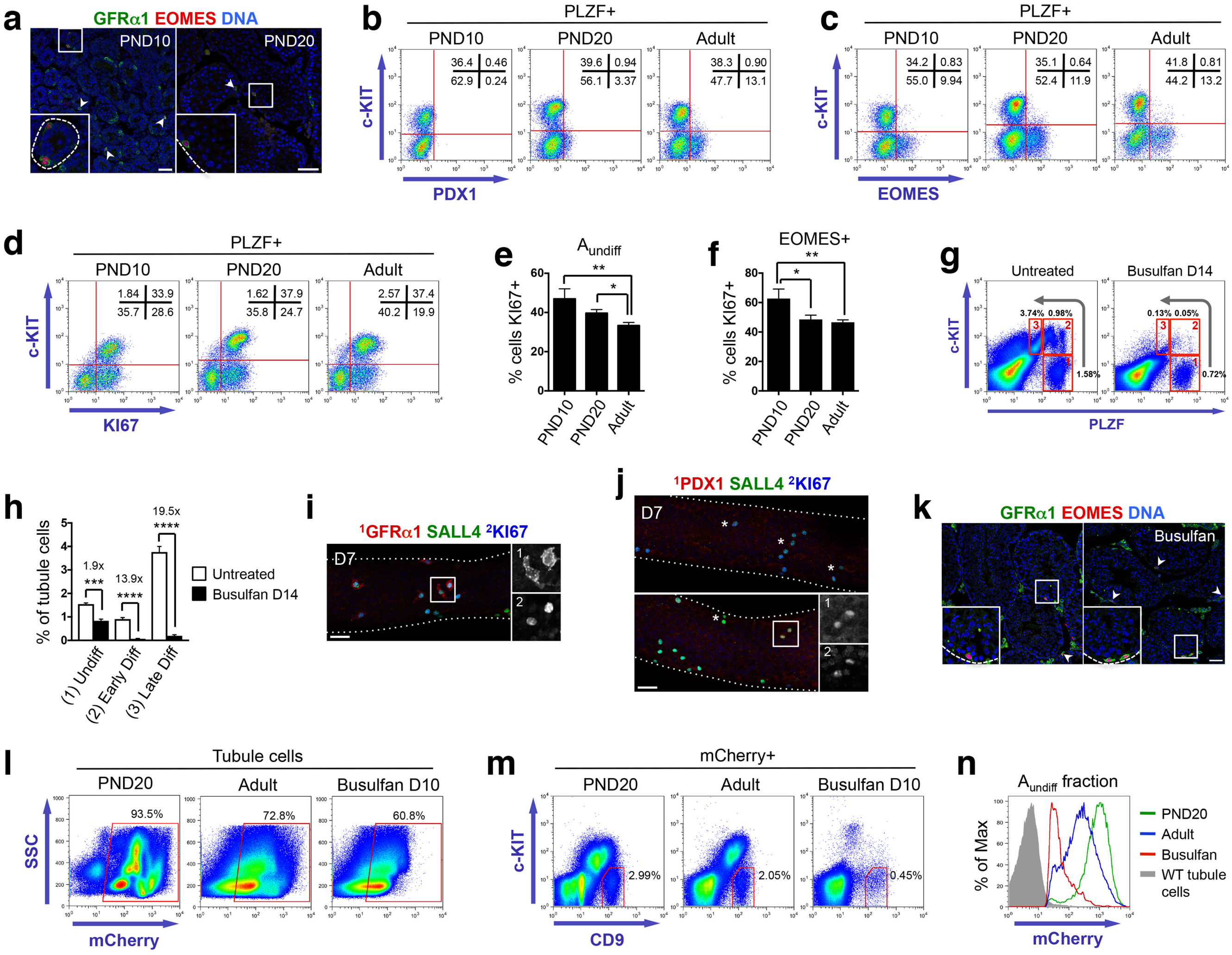

Supplementary Figure 7. Characterization of PDX1+ and EOMES+ A_{undiff} populations. (**a**) Representative IF of adult wildtype (WT) testis sections of indicated postnatal ages (n=3 mice per timepoint). Insets show selected areas at higher magnification. *Eomes* expression is restricted to the GFRα1+ spermatogonial pool at all ages (selected cells indicated with arrowheads). Scale bar, 50 µm. (**b**-**d**) Representative flow cytometry of fixed and permeabilized testis cells from WT mice of indicated ages (postnatal day; PND). PLZF+ cell population is shown. Percentage of cells within respective gates is indicated. n=3-4 PND10 mice, n=4 PND20 and n=4-5 adults. (**e**) Quantification of flow cytometry from **d**. Mean percentages of PLZF+ c-KIT– cells (A_{undiff}) positive for KI67 ± s.e.m. are shown. (**f**) Data from flow cytometry as in **d**. Mean percentages of PLZF+ EOMES+ cells positive for KI67 ± s.e.m. are shown. (**g**) Representative flow cytometry of fixed and permeabilized testis cells from WT mice treated with busulfan (10mg/kg) and analyzed after 14 days (D). Untreated controls are shown. Populations #1 are undifferentiated cells (PLZF+ c-KIT–), #2 early differentiating cells (PLZF+ c-KIT+) and #3 late differentiating cells ($PLZF^{low}$ c-KIT+). Percentage of cells within respective gates is indicated. 4 mice per condition were analyzed. (**h**) Quantification of flow cytometry from **g**. Graph shows mean percentage of cells in each population ± s.e.m. (n=4 mice per condition). (**i**) Testes from duplicate WT adults treated with busulfan as in **g** were harvested at indicated timepoints after treatment (D=days) and analyzed by wholemount IF. Insets show single immunostaining of indicated region at higher magnification. Scale bar, 50 µm. (**j**) Regeneration assay of **i**. Few remaining A_{undiff} D7 after treatment expressed *Pdx1* indicating that the PDX1+ population is busulfan-sensitive. A rare PDX1+ A_{pr} is shown in lower panels. Asterisks: PDX1– A_{undiff}. Scale bar, 50 µm. (**k**) Representative IF of testis sections from WT adults treated with busulfan as in **g** or untreated controls (n=2 mice). Insets show selected areas at higher magnification. *Eomes* expression is restricted to the GFRα1+ spermatogonial pool following busulfan (selected cells indicated with arrowheads). Scale bar, 50 µm. (**l**-**n**)

Representative flow cytometry of live testis cells from Plzf-mC/CreER mice of indicated postnatal ages or 10 days (D) after treatment with busulfan as in **g** (n=5 PND20, n=6 adults, n=4 busulfan-treated adults). SSC is side scatter. Percentages of cells within respective gates are indicated. mCherry+ gate was set according to WT control. mCherry+ cells are shown in **m**. Overlay histogram in **n** illustrates relative levels of mCherry in gated A_{undiff} population (mCherry+ CD9+ c-KIT–) *vs.* WT tubule cells. Significance was calculated by two-tailed Student's *t*-test (*P < 0.05, **P < 0.01, ***P < 0.001, ****P < 0.0001).

Supplementary Table 1 Primer sequences for qRT-PCR

Gene	Primer Sequence	
	**Forward**	**Reverse**
Actin	GGCTGTATTCCCCTCCATCG	CCAGTTGGTAACAATGCCATGT
Ddit4	CAAGGCAAGAGCTGCCATAG	CCGGTACTTAGCGTCAGGG
Dmrt2	ATTTCGATCGGAAAGCAGTG	TCCTTGTCAGCAAAGGCTCT
Dnmt3l	GTTCCTGGAGTCCCTCTTCC	CCATGGCATTGATCCTCTCT
Dppa3	ATCCGGAGGGAAGTTCAAAG	CACTGTCCCGTTCAAACTCA
Egr2	AGCTGCCTGACAGCCTCTAC	GGAGATCCAGGGGTCTCTTC
Eomes	GGCAAAGCGGACAATAACAT	AGCCTCGGTTGGTATTTGTG
Esr1	AATAGCCCTGCCTTGTCCTT	AGGTGGACCTGATCATGGAG
Etv4	TGAAAGGCGGATACTTGGAC	TGTCCGGTACCTGAGCTTCT
Etv5	CGAGAGACTGGAAGGCAAAG	TGGCCGATTCTTCTGGATAC
Gfi1	CCTGGTCAAGAGCAAGAAGG	CCTCGGTAAGCTGAGAGTCG
Gfra1	CACTCCTGGATTTGCTGATGT	AGTGTGCGGTACTTGGTGC
Id4	CAGTGCGATATGAACGACTGC	GACTTTCTTGTTGGGCGGGAT
Ifitm1	GGTCACCCCACATCTCAACT	CCACCATCTTCCTGTCCCTA
Kit	GCCACGTCTCAGCCATCTG	GTCGGGATCAATGCACGTCA
Lhx1	CAGTGTCGCCAAAGAGAACA	TCAACGTCTCCAGTTGCTTG
Lhx2	CCAGCTTCGGACAATGAAGT	TTTCCTGCCGTAAAAGGTTG
Lin28a	GGCATCTGTAAGTGGTTCAACG	GCCAGTGACACGGATGGATT
Nanos2	ATTCAGAGCCGGAAGCAAAG	GACTGCTGTTGAGTGGACAA
Nanos3	ATGGGGACTTTCAATCTTTGGAC	GTTTGCAGAATGAACATAAGCGT
Ngn3	GCTATCCACTGCTGCTTGA	CCGGGAAAAGGTTGTTGTGT
Onecut2	AGAGGGTTCTATGCCGGTCT	GGGATTTCTTCTGCGAGTTG
Pdx1	GGACATCTCCCCATACGAAG	GTTCCGCTGTGTAAGCACCT
Plzf	CTCCGTAAGCGTCCCCTCTGC	GGTGCAGGCTAGCACCGTCC
Pou5f1	CAGCCAGACCACCATCTGTC	GTCTCCGATTTGCATATCTCCTG
Rarg	GGGCAAGTACACCACGAACT	ATCCGCAGCATTAGGATGTC
Ret	GCATGTCAGACCCGAACTGG	CGCTGAGGGTGAAACCATCC
Sall4	CCCTGGGAACCTGCGATGAAG	TCAGAGAGACTAAAGAACTCGGC
Smad6	CCACCAACTCCCTCATCACT	CTGCCCTGAGGTAGGTCGTA
Snai1	CTTGTGTCTGCACGACCTGT	CTTCACATCCGAGTGGGTTT
Sohlh1	GGCATCTGTCCTGGAGATGT	CACAGCAGATGGTTTGGCTA
Sox10	TAGCCGACCAGTACCCTCAC	TGTAGTCCGGATGGTCCTTT
Sox3	ACGCATCAGGTGAGAGAAGC	CGGGGTTCTTGAGTTCAGTC
Stra8	ACAACCTAAGGAAGGCAGTTTAC	GACCTCCTCTAAGCTGTTGGG
T	GAACCTCGGATTCACATCGT	TTCTTTGGCATCAAGGAAGG
Tcl1	GATCTGGGAGAAGCACGTGTA	CCACATTAAAGGCAGCTCGT
Tdh	AAGCTTGTCTTGCCCTTGAA	CTTCCCAAATCGTTTCCTCA
Upp1	GGTTCAGGAGTTGGTGCAGT	GCAAACACCGAAGATTCCAT
Utf1	ATAACCAGATCCGCCAACTC	TTCGTCGTGGAAGAACTGAA
Vasa	TGTGCCACAACTTCTGAGGC	CCTGATTTCGGTTTCATCCATCC
Zfp467	CTCCTTGGGATTCTCAGTGG	GCCCATACAAGGAGCTTCAG
Zic5	GTCTGCTTTTGGGAGGACTG	ATCGCTGCTATTGGCAAACT
