# Supplementary material for: Identification of dynamic undifferentiated cell states within the male germline
Source: Nat Commun. 2018 Jul 19;9:2819. doi: 10.1038/s41467-018-04827-z (PMC6053434; doi:10.1038/s41467-018-04827-z)
Supplement: Supplementary file 2 — Description of Additional Supplementary Files [file 41467_2018_4827_MOESM2_ESM.docx]

**Description of Additional Supplementary Files**

File Name: Supplementary Data 1

Description:

**Gene expression analysis of Oct4-GFP+ and Oct4-GFP– A_undiff_.** Testis cell fractions were sorted from pooled Plzf-mC/CreER; Oct4-GFP adults and gene expression analyzed by microarray (n=3 independent sorts). Expression of genes enriched in Oct4-GFP– and GFP+ fractions are included as separate sheets. FC is fold change. Expression of a panel of genes identified from the microarray plus other germ cell-related and control genes were validated by qRT-PCR in independent sorted samples of Oct4-GFP+ and Oct4-GFP– A_undiff_ and in GFP+ and GFP– fractions of cultured spermatogonia established from equivalent A_undiff_ populations from Oct4-GFP; Plzf-mC/CreER adults. The list of these analyzed genes is included in the separate excel sheet. The A_undiff_ fraction that expression of the gene of interest was enriched in is indicated. N/A indicates genes not identified as differentially expressed by microarray.

File Name: Supplementary Data 2

Description:

**KEGG pathway analysis of differentially expressed genes (DEG) in PDX1+ and PDX1– A_undiff_.** GFP+ and GFP– A_undiff_ fractions from Plzf-mC/CreER; *Pdx1*^GFP/+^ adults (n=4 mice) were analysed by RNA-Seq. DEG between the populations (cut-off is False discovery rate < 0.05 and absolute fold change ≥ 1.5) were subjected to KEGG pathway analysis. Table lists significant identified pathways (P < 0.05).
